# Pandemic-related emergency psychiatric presentations for self-harm of children and adolescents in 10 countries (PREP-kids): a retrospective international cohort study

**DOI:** 10.1007/s00787-021-01741-6

**Published:** 2021-03-07

**Authors:** Dennis Ougrin, Ben Hoi-ching Wong, Mehrak Vaezinejad, Paul L. Plener, Tauseef Mehdi, Liana Romaniuk, Elizabeth Barrett, Haseena Hussain, Alexandra Lloyd, Jovanka Tolmac, Manish Rao, Sulagna Chakrabarti, Sara Carucci, Omer S. Moghraby, Rachel Elvins, Farah Rozali, Ereni Skouta, Fiona McNicholas, Natalie Kuruppuaracchi, Dejan Stevanovic, Peter Nagy, Chiara Davico, Hassan Mirza, Evren Tufan, Fatima Youssef, Ben Meadowcroft, Sabine Landau

**Affiliations:** 1 Child and Adolescent Psychiatry, Kings College London, London, UK; 2 South London and Maudsley Mental Health NHS Trust, London, UK; 3 Medical University of Vienna, Vienna, Austria; 4 Berkshire Healthcare NHS Foundation Trust, Bracknell, Bracknell Forest, UK; 5 The University of Edinburgh Centre for Clinical Brain Sciences, Edinburgh, UK; 6 Temple Street Children’s University Hospital, Dublin, Ireland; 7 Hertfordshire Partnership University NHS Foundation Trust, Child and Adolescent Mental Health Services, Hatfield, Hertfordshire UK; 8 Central and North West London NHS Foundation Trust, London, UK; 9 Università Degli Studi Di Cagliari Facoltà Di Medicina E Chirurgia Monserrato, Sardegna, Italy; 10 Manchester University NHS Foundation Trust, Greater Manchester, UK; 11 NHS Lothian, Edinburgh, UK; 12 University College Dublin School of Medicine, Dublin, Ireland; 13 Clinic for Neurology and Psychiatry for Children and Youth, Belgrade, Serbia; 14 Bethesda Children’s Hospital, Budapest, Hungary; 15 Universita Degli Studi Di Torino, Turin, Italy; 16 Sultan Qaboos University, Muscat, Oman; 17 Abant Izzet Baysal University Medical Faculty, Bolu, Turkey; 18 Dubai Department of Medical Education, Dubai, United Arab Emirates; 19 King’s College London, Biostatistics, London, UK; 20 Department of Child and Adolescent Psychiatry and Psychotherapy, University of Ulm, Ulm, Germany; 21 Vadaskert Child and Adolescent Psychiatric Hospital, Vadaskert, Hungary

**Keywords:** Self-harm, COVID-19, Pandemic, Emergency presentation, Children, Adolescents

## Abstract

To examine the differences in hospital emergency psychiatric presentations for self-harm of children and adolescents during the covid-19 lockdown in March and April 2020 compared with the same period in 2019. Retrospective cohort study. We used electronic patient records from 23 hospital emergency departments in ten countries grouped into 14 areas. We examined data on 2073 acute hospital presentations by 1795 unique children and adolescents through age 18. We examined the total number of emergency psychiatric hospital presentations and the proportion of children and adolescents presenting with severe self-harm as our two main outcome measures. In addition, we examined sociodemographic and clinical characteristics and clinical management variables for those presenting with self-harm. To compare the number of hospital presentations between 2020 and 2019 a negative binomial model was used. For other variables, individual participant data (IPD) meta-analyses were carried out. Emergency psychiatric hospital presentations decreased from 1239 in 2019 to 834 in 2020, incident rate ratio 0.67, 95% CI 0.62–0.73; *p* < 0.001. The proportion of children and adolescents presenting with self-harm increased from 50% in 2019 to 57% in 2020, odds ratio 1.33, 1.07–1.64; *p* = 0.009 but there was no difference in the proportion presenting with severe self-harm. Within the subpopulation presenting with self-harm the proportion of children and adolescents presenting with emotional disorders increased from 58 to 66%, odds ratio 1.58, 1.06–2.36; *p* = 0.025. The proportion of children and adolescents admitted to an observation ward also decreased from 13 to 9% in 2020, odds ratio 0.52, 0.28–0.96; *p* = 0.036. Service planners should consider that, during a lockdown, there are likely to be fewer emergency psychiatric presentations. Many children and adolescents with psychiatric emergencies might not receive any service. A focus on developing intensive community care services with outreach capabilities should be prioritised.

**Supplementary Information:**

The online version contains supplementary material available at 10.1007/s00787-021-01741-6.

## Introduction

Suicide is the second leading cause of death in children and adolescents in most developed countries, exceeded only by accidents [1]. Self-harm, necessitating hospital treatment, is the strongest predictor of suicide in this population, particularly if medical intervention is required [2]. Both suicide and self-harm in children and adolescents have been rising in the UK and other countries [3]. Hospital presentations to emergency departments with self-harm have seen a particularly sharp increase, although the number of children and adolescents seeking help for self-harm has also increased in primary care [4]. There has also been an increase in the number of children and adolescents reporting self-harm in surveys of the general population, especially older girls [5]. These increases in self-harm have been paralleled by increases in suicide in older teenagers (15–19-year-olds), rising from 4.1 to 6.7/100,000 between 2010 and 2018 [6].

There has been some progress in the treatment of young people with self-harm [7, 8], but many challenges remain [9]. Post mortem analyses following youth suicide suggest that up to 40% had no prior mental health access [10], and at least three quarters of children and adolescents with self-harm do not access any health services [11]. However, severe self-harm is a common cause of inpatient admissions. There has been a significant increase in the number of psychiatric inpatient admissions in England, from approximately 2200 to 4500 between 2008 and 2018 which then plateaued and started to decrease in 2019 [12]. The reasons for this recent reduction are complex, however, a rapid expansion of intensive community care services may have been a deciding factor [13–15]. Lengthy inpatient admissions for children and adolescents with self-harm, although sometimes needed, have been linked with an increase in self-harm during admissions [16]. Alternatives to inpatient admissions exist, but their evaluation is still in its infancy [17].

The outbreak of Covid-19 and the associated quarantine (lockdown) measures are likely to have had a substantial influence on children and adolescents’ mental health. Previous epidemics have been associated with an increase in emotional disorders and neuropsychiatric disorders [18], although the impact of these epidemics has been poorly studied outside of the most affected nations [19].

The impact of the outbreak of the novel coronavirus disease 2019 (covid-19) on children and adolescents’ mental health remains poorly studied [18]. The pandemic started in China, in December 2019. As of the 4th of August 2020, 18 million people have contracted the disease and nearly 700,000 people have died. The actual number of people infected and diseased is likely to have been substantially higher due to poor availability of testing. The key milestone of the quarantine in the UK with likely impact on children and adolescents’ mental health in March 2020 included the following: the announcement of the Government’s Coronavirus Action Plan (03/03) including advice to go to hospital only when absolutely necessary; cancellation of school trips abroad (12/03), advice on cancelling all non-essential trips (16/03), an announcement of school closures (18/03) and freedom of movement restrictions enforceable by law (23/03) www.gov.uk/coronavirus before gradual easing in May 2020. Most other countries instituted similar measures in March 2020. Full or partial school closures were implemented in all European countries except Belarus by 18/03.

The impact of the outbreak and these measures on children and adolescents’ emergency psychiatric presentations is unknown. Yet, it is crucial to understand this impact to prepare the services for any future drastic reductions in children and adolescents’ social, educational and leisure activities.

To assess the impact of the Covid-19 outbreak and these measures on children and adolescents’ hospital presentations with self-harm and other mental health emergencies we established a network of clinicians, researchers and children and adolescents with lived experience of emergency service use in ten countries. We used two existing networks of researchers, the European College of Neuropsychopharmacology (ECNP) Research & Scholarship Foundation and the Comparison of Effectiveness and Cost-Effectiveness of Intensive Community Care Services versus Usual Inpatient Care for Young People with Psychiatric Emergencies (IVY). 23 individual emergency hospital departments in ten countries participated, divided into 14 areas based on sociodemographic and geographic similarities of hospital catchment areas. Most of the included countries had seen consistent increase in hospital emergency presentations for self-harm for many years pre-Covid-19.

To evaluate the impact of the outbreak and the associated quarantine measures, we used local electronic patient data to investigate the hospital presentations in detail, comparing sociodemographic and clinical characteristics of the presentations between March and April 2020 and March and April 2019. Clinical management patterns were also included. For example, follow-up appointments are required by NICE as the first week after a self-harm presentation carries the highest risk of suicide.

Based on routine data from one included London paediatric emergency department available at the planning stage, we hypothesised, that the total number of emergency presentations will be lower in March and April 2020 versus March and April 2019, but the proportion of children and adolescents with severe self-harm will be higher. To the best of our knowledge, this is the first report investigating the impact of the Covid-19 pandemic on emergency mental health presentations of children and adolescents.

## Methods

### Study design and population

Children and adolescents aged through 18 years who presented to hospital emergency departments with self-harm and other mental health emergencies during March–April 2020 or March–April 2019 were included in this study. The study uses a retrospective cohort study design to compare the 2020 and 2019 cohorts in terms of characteristics of emergency presentations.

### Data sources

Electronic patient records of 23 hospital emergency departments in 10 countries (England, Scotland, Ireland, Austria, Italy, Hungary, Serbia, Turkey, Oman and the United Arab Emirates) subdivided into 14 relatively geographically homogeneous areas: London, Dublin, Edinburgh, Livingstone, Manchester, the Home Counties (suburban areas around London), Turin, Cagliari, Vienna, Budapest, Belgrade, Istanbul, Muscat and Dubai. The hospital emergency departments serve the total population of approximately 31.2 million with 6.5 million under-18 s. There is a total of approximately 200,000 paediatric emergency presentations per year. The hospitals are representative of health care systems in developed high-income (England, Scotland, Ireland, Austria, Italy, Hungary), developing middle-income (Serbia and Turkey) and developing high-income (Oman and the United Arab Emirates) countries.

### Outcomes

#### All presentations

We measured the following sociodemographic characteristics for the entire sample of presentations: sex, age, dominant ethnic group (yes/no), decile of deprivation index, young person in education, employment or training (yes/no), young person looked after by the local authority and the young person’s biological parents living together out of those who are looked after by their parents (yes/no). We then measured the following clinical characteristics for all emergency presentations: presentation for self-harm (yes/no), children and adolescents with previous hospital presentations for self-harm (yes/no), or with previous self-harm in the community (yes/no).

#### Sub-sample with self-harm presentations

For emergency presentations for self-harm we measured sociodemographic characteristics; clinical characteristics, including proximal risk factors for self-harm, clinical diagnosis and distal risk factors for self-harm; clinical management variables. The following clinical variables were measures: severe self-harm (yes/no), presence (yes/no) of each of emotional disorders, behavioural disorders, psychotic disorders, eating disorders, neurodevelopmental disorders, substance misuse disorder, somatoform disorders or personality disorders, use of a violent method of self-harm (yes/no) and suicide intent (yes/no). We then assessed whether or not a row with a family member or social isolation were precipitants of self-harm, whether the children and adolescents used social media to communicate about self-harm, used alcohol at the time of self-harm, had previous hospital emergency presentation for self-harm, had previous hospital emergency presentation for reasons other than self-harm, had previous self-harm in the community, previous psychiatric inpatient treatment and had a family history of self-harm. Finally, we measured the following variables reflecting patterns of clinical management of self-harm: young person detained under a section of Mental Health Act (yes/no), length of stay in the emergency department, dichotomised to more or less than 24 h; admission to observation wards in emergency departments (yes/no), acute wards (yes/no), Intensive Treatment Units (yes/no), psychiatric inpatient wards (yes/no); offer of community follow-up within seven days of the presentation with self-harm (yes/no) and attending at least one follow-appointment within seven days of the original hospital presentation if community follow-up was offered (yes/no). We recorded phone, remote or face-to-face follow-up appointments.

Self-harm was defined using the UK National Institute for Health and Care Excellence clinical guidelines as “any act of self-poisoning or self-injury, irrespective of the underlying intent” (NICE), thus incorporating both non-suicidal self-injury, suicide attempts non-suicidal self-poisoning and self-harm with unclear or mixed intent. Severe self-harm was defined as meeting at least one of the following criteria: (1) A high-lethality method used (drowning, hanging, jumping from heights, shooting, potentially lethal self-poisoning in the absence of medical care and self-injury involving major vessels) (2) Any self-harm resulting in an Intensive Care Unit admission 3. Any self-harm resulting in an admission to an acute ward for 72 h or more.

### Sample size calculation

The two main outcomes of interest in this study were (a) frequency of hospital presentation and (b) proportion of presentations with severe self-harm out of all hospital presentations for self-harm. To address (a) we simply collected presentations from as many different areas as possible within the available data collection period. To address (b) we used evidence from one London emergency department obtained in March 2020 that suggested that while the number of presentations might be reduced to a quarter the number in 2019, within those with presentations for self-harm the proportion of severe self-harm might have increased from around 8% in 2019 to around 40% in 2020. Based on a two-sided Fisher’s exact test with significance level 5% and a power requirement of 90% we calculated that a minimum of 84 self-harm presentations in 2019 and 21 self-harm presentations in 2020 would be required to detect such an effect within a hospital area.

### Statistical analysis

Demographic and clinical characteristics of the sample of hospital emergency presentations were described using relevant summary statistics within site and year, and overall. The formal statistical analyses assessed the effect of the pandemic/interventions by comparing 2020 outcomes with 2019 outcomes for each site and across areas.

To compare the number of hospital emergency presentations between 2020 and 2019 a negative binomial model was fitted to the 28 counts of hospital emergency presentations from 14 areas in two years. The negative binomial model was chosen to allow for a positively skewed distribution of the counts as well as over-dispersion, e.g., because hospital emergency presentations were by the same young person. The model contained the hospital emergency presentations count as the dependent variable and year and hospital area as explanatory variables.

To compare (1) proportions of children and adolescents presenting with self-harm, having previously presented with self-harm to a hospital or engaged in self-harm in the community, and (2) the characteristics of those presenting at hospital for self-harm between 2020 and 2019, individual participant data (IPD) meta-analyses will be used. The modelling proceeded in two stages: In the first stage relevant linear or logistic regression models were fitted for each area separately. These models contained the respective outcome as the dependent variable and year (2020 vs 2019) as the explanatory variable. In the second stage, relevant site-specific effects were combined into an estimate of an overall year effect using meta-analysis. We opted for a random effects meta-analysis approach to combine effect size estimates. To account for the correlation between repeated outcome measures from the same young person cluster bootstrapping was used to generate 95% confidence interval with the resampling cluster defined by the person (1000 bootstrap samples). When modelling binary outcomes areas with cell counts of less than five in the cross-tabulation of the outcome by year were excluded from the IPD meta-analysis to ensure that bootstrapping inferences were reliable. We report the overall year effect estimate unless there is evidence for effect modification by area according to Cochran’s *Q* test. A measure of the amount of between-area year effect variability is provided by the *I*^2^ statistic, and which is shown on the resulting forest plots. The analyses were implemented in Stata using the command ipdmetan [20].

Where (1) self-harm variables or (2) other outcome variables in those who presented with self-harm were missing presentations were excluded from the area-level and overall year comparison. In other words, complete case analyses were used which assumed that within years observations were missing completely at random.

## Results

Records from a total of 2073 hospital emergency presentations by 1795 unique children and adolescents from 14 areas (23 hospitals) during March/April 2020 (*n* = 834) and March/April 2019 (*n* = 1239) were collected. See supplementary materials for hospital characteristics.

### All hospital emergency presentations (the whole sample)

The number of emergency presentations by area and year is shown in Table 1. All areas recorded fewer hospital presentations over the same period in 2020 compared to 2019. This reduction was statistically significant. The incidence rate ratio was estimated as 0.67, 95% confidence interval 0.62–0.73; *p* < 0.001.

**Table 1:** Number of hospital emergency presentations by area and year

Area	2019	2020	Total
London	261	157	418
Manchester	46	28	74
Home counties	304	217	521
Livingstone	83	55	138
Edinburgh	87	48	135
Dublin	96	86	182
Cagliari	53	27	80
Turin	15	13	28
Vienna	211	160	371
Budapest	21	9	30
Belgrade	22	8	30
Istanbul	12	10	22
Muscat	15	12	27
Dubai	13	4	17
Total	1239	834	2073

### Sociodemographic characteristics of all hospital emergency presentations

Those presenting at hospital emergency departments were mostly female in all areas (67.5% female, 30.7% male and 1.8% other), with an average age of 15 years. About three quarters were from the dominant ethnic group. The overall deprivation decile for English and Scottish areas was 5.5 with variation across areas (lowest average decile 2.7 for Manchester, highest 6.9 for the home counties). For the 1290 presentations where this information was available, 89% were in education, employment or training. For the 1352 presentations for whom this was known, 12% were Looked After Children and Adolescents (LAC), looked after by the local authority. There was considerable variability across areas, with Dubai and Budapest having no LAC while for Istanbul and Cagliari this proportion was more than 20%. Of those who were not LAC, 41% had biological parents who were living together.

### Self-harm variables in all hospital emergency presentations

**Fig. 1:**
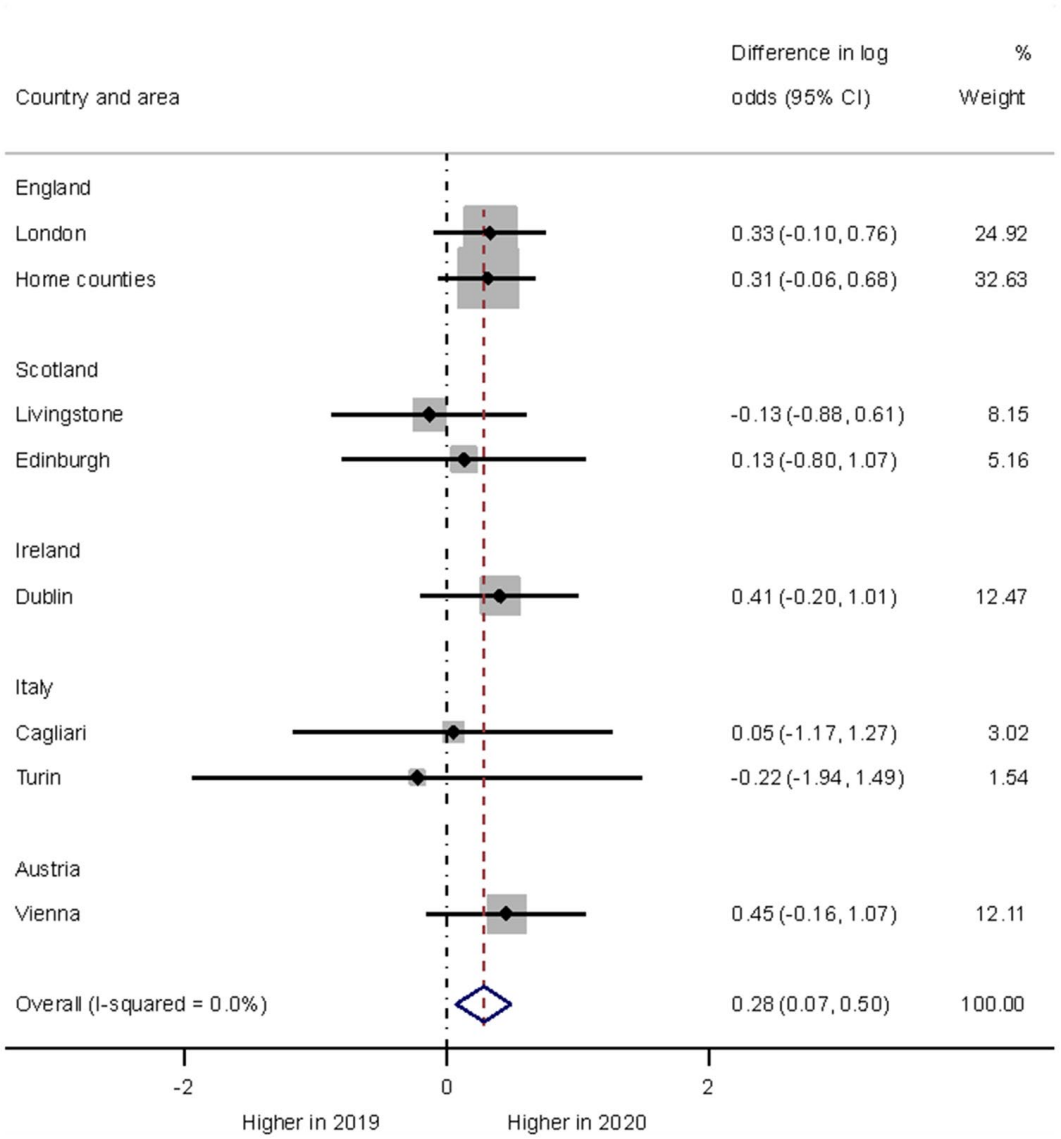
Forest plot illustrating year differences in hospital self-harm presentations

Overall, the proportion of children and adolescents presenting with self-harm at hospital emergency departments was about half (1082 presentations) of all presentations and was higher in 2020 (57%) than in 2019 (50%). The formal year comparison of the proportions of children and adolescents presenting at hospital emergency departments with self-harm was statistically significant (*p* = 0.009).The negative binomial model estimated a 33% increase in odds in 2020 (odds ratio 1.33, 95% confidence interval 1.07–1.64). The forest plot in Fig. 1 shows the area-specific year effects. 1845 presentations from eight areas contributed to the formal analysis. While there was some between-area variability in effects there was no indication of effect size modification by area (*I*^2^ < 0.1) and thus assessing the average difference between years seemed warranted.

A history of previous self-harm was recorded for many children and adolescents seen at hospital emergency departments. Overall, the proportions of children and adolescents who previously presented at hospital for self-harm or had self-harmed in the community were higher in 2020 (increased from 29 to 36%, and from 63 to 71% respectively). Based on the eight areas for which we had sufficient numbers to attempt formal analyses, the proportion of children and adolescents with a history of a previous hospital presentation for self-harm was significantly increased in 2020 (odds ratio 1.40, 95% confidence interval 1.05–1.87; *p* = 0.022). The observed increase in the proportion of children and adolescents with a previous history of self-harm in the community did not test statistically significant based on the seven areas that provided sufficient data (*p* = 0.16). Thus, within those attending hospital emergency departments in 2020, there was a preponderance of children and adolescents presenting with self-harm and an increase in those children and adolescents with a history of previous hospital presentations for self-harm.

### Children and adolescents presenting with self-harm (a sub-sample)

There were 1082 hospital presentations for self-harm from 952 unique children and adolescents. Table 2 summarizes the findings of sub-sample analyses.

**Table 2:** Characteristics, risk factors, and clinical management of self-harm presentations (*n* = 1082 events)

	Descriptive summaries			Formal analyses based on areas with sufficient data^a^		
	Proportion (available sample size) unless otherwise indicated			Estimates comparing 2020 with 2019	95% CI	*p* value
	2019 (*n* = 612)		2020 (*n* = 470)			
*Sociodemographic characteristics*						
Female	72% (601)	75% (462)		OR = 1.28	(0.89 to 1.86)	0.18
Age	*M* = 15.2, SD = 2.0 (612)	*M* = 15.4, SD = 1.7 (466)		Mean difference = 0.24	(− 0.01 to 0.50)	0.064
Dominant ethnic group	73% (520)	74% (375)		OR = 0.95	(0.53 to 1.68)	0.85
Deprivation decile	*M* = 5.4, SD = 2.7 (439)	*M* = 5.6, SD = 2.9 (321)		Mean difference = 0.18	(− 0.24 to 0.60)	0.40
In EET	88% (442)	88% (306)		OR = 0.75	(0.26 to 2.20)	0.60
Looked after by local authority	0.15 (465)	0.13 (325)		OR = 0.88	(0.44 to 1.76)	0.72
Parents living together	38% (342)	41% (221)		OR = 1.05	(0.64 to 1.73)	0.85
*Proximal risk factors and clinical diagnoses*						
Severe self-harm	19% (605)	19% (469)		OR = 1.22	(0.72 to 2.07)	0.47
Emotional disorders	58% (520)	66% (384)		OR = 1.58	(1.06 to 2.36)	0.025
Behavioural disorders	13% (520)	14% (384)		OR = 1.09	(0.56 to 2.14)	0.80
Psychotic disorders	2% (520)	3% (384)		n/a	n/a	n/a
Eating disorders	4% (520)	4% (384)		n/a	n/a	n/a
Neurodevelopmental disorders	14% (520)	16% (384)		OR = 1.40	(0.87 to 2.25)	0.16
Substance misuse disorders	7% (520)	7% (384)		OR = 2.00	(0.62 to 6.45)	0.25
Somatoform disorders	8% (520)	2% (384)		n/a	n/a	n/a
Personality disorders	16% (520)	14% (384)		OR = 0.77	(0.41 to 1.47)	0.43
Violent method of self-harm	6% (599)	8% (461)		OR = 1.55	(0.56 to 4.31)	0.40
Suicide intent in those with self-harm	49% (592)	55% (435)		OR = 1.48	(0.99 to 2.21)	0.057
Row with family a precipitant	32% (441)	38% (308)		OR = 1.31	(0.76 to 2.25)	0.33
Social isolation a precipitant	9% (441)	17% (308)		OR = 1.59	(0.80 to 3.18)	0.19
Communicating about self-harm on social media	7% (428)	8% (304)		n/a	n/a	n/a
Alcohol involved in self-harm	8% (508)	10% (373)		OR = 1.42	(0.70 to 2.89)	0.33
*Distal risk factors*						
Previous hospital presentation for self-harm^b^	38% (465)	47% (324)		OR = 1.42	(0.97 to 2.08)	0.073
Previous hospital psychiatric presentation other than for self-harm	22% (449)	24% (272)		OR = 1.07	(0.62 to 1.85)	0.80
Previous self-harm in the community^b^	76% (467)	81% (341)		OR = 1.19^c^	(0.58 to 2.44)	0.632
Psychiatric inpatient treatment in the past year	15% (466)	18% (329)		OR = 1.24	(0.59 to 2.61)	0.57
Psychiatric inpatient treatment in the past month	6% (466)	5% (329)		n/a	n/a	n/a
Psychiatric inpatient treatment in the past week	2% (469)	3% (329)		n/a	n/a	n/a
Family history of self-harm	20% (204)	18% (196)		OR = 1.45	(0.43 to 4.86)	0.55
*Clinical management*						
Detained under a Mental Health Act	3% (411)	3% (320)		OR = 1.00	(0.26 to 3.75)	1.0
Staying in hospital one day or longer	27% (536)	32% (414)		OR = 1.30	(0.93 to 1.80)	0.12
Admitted to an observation ward	13% (548)	9% (422)		OR = 0.52	(0.28 to 0.96)	0.036
Admitted to an acute ward	18% (547)	20% (422)		OR = 1.30	(0.58 to 2.87)	0.53
Admitted to an ITU	1% (547)	1% (422)		n/a	n/a	n/a
Admitted to a psychiatric inpatient ward	14% (592)	16% (468)		OR = 1.73	(0.73 to 4.08)	0.21
Offered a follow-up appointment	71% (479)	77% (371)		OR = 1.21	(0.63 to 2.32)	0.57
Attended a follow-up appointment	86% (310)	89% (238)		OR = 1.36	(0.60 to 3.1)	0.46

Includes multiple presentations from the same children and adolescents (952 different children and adolescents)

^a^Presentations from areas with too low cell counts in the binary outcome x year cross-tabulation cannot contribute to the formal year comparison, represented with n/a, not applicable

^b^The figures refer only to those young people with self-harm presentations, compare with the figures for the entire sample in the relevant section above

^c^There was a statistically significant effect heterogeneity in year effects between areas (Cochran’s *Q* test = 0.023, I^2^ = 61.5%)

*M* mean, *SD* standard deviation, *OR* odds ratio, *CI* confidence interval, *EET* education, employment or training, *n/a* not applicable

### Sociodemographic characteristics

Children and adolescents presenting for self-harm were mainly female, on average 15 years old, tended to be from the dominant ethnic group in the country, tended to be in education, employment or training and not looked after by the local authority. The largest observed year difference was in the proportion of females. However, neither this nor any other year difference in demographic variables of this subpopulation tested statistically significant. There was, therefore, no evidence to suggest that demographic characteristics were affecting the presentations of children and adolescents with self-harm after the outbreak of the pandemic.

### Clinical characteristics

We considered two groups of clinical characteristics: proximal risk factors and clinical diagnosis; and distal risk factors. This categorisation is well established in research and is used by many clinicians [21, 22].

To establish the reliability of self-harm severity ratings, we selected 10% of the notes from the 2020 data and calculated the interrater agreement. There was an excellent interrater agreement, *κ*  = 0.91, standard error of *κ* = 0.05, 95% confidence interval 0.82 to 0.99.

**Fig. 2:**
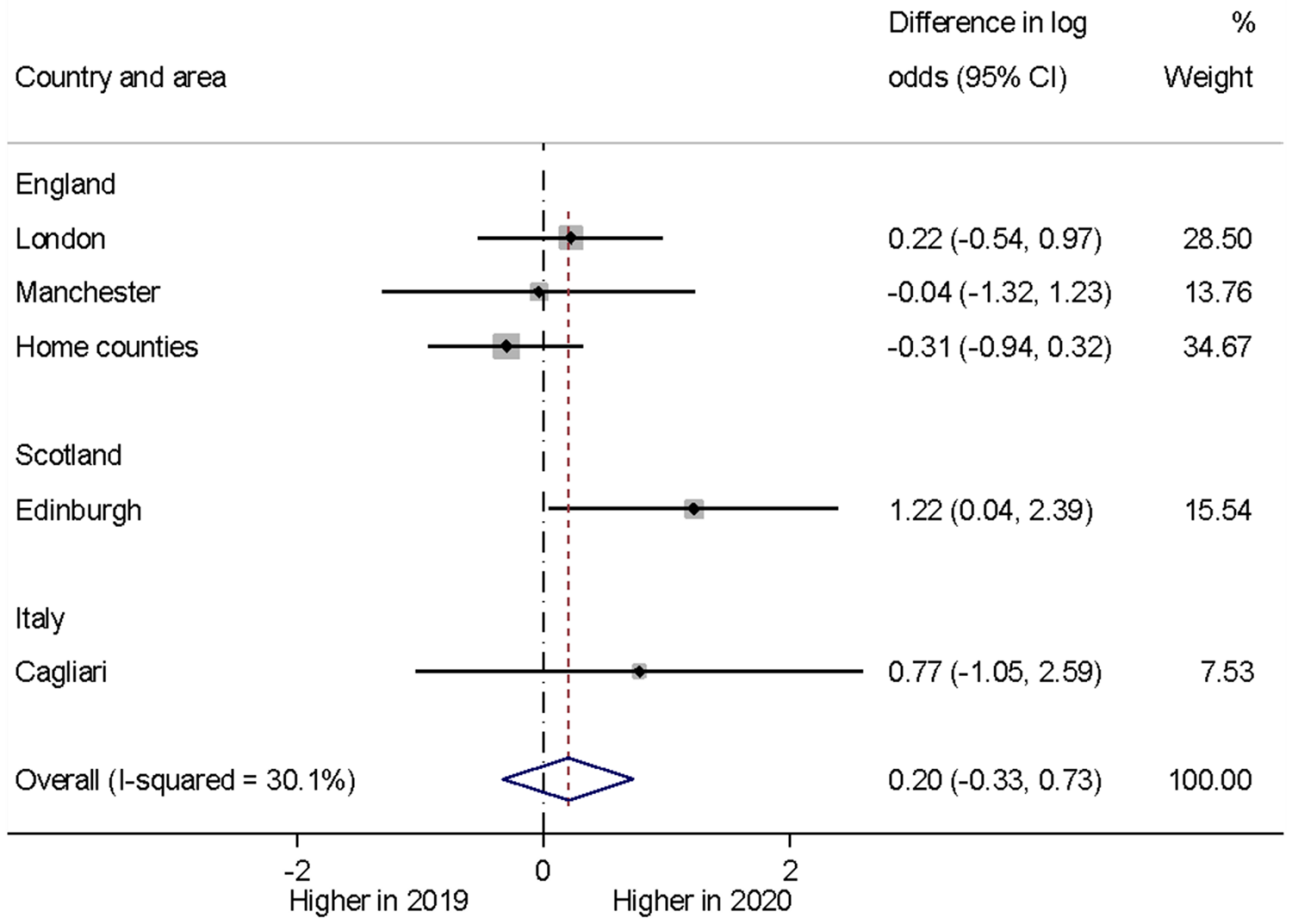
Forest plot illustrating year differences in severe self-harm presentations)

We did not find any evidence for a year difference in the proportion of the children and adolescents with severe self-harm presentations based on the five areas (with 717 presentations) that contributed sufficient data to assess this hypothesis formally (*p* = 0.47). While the forest plot in Fig. 2 shows an apparent increase in the frequency of severe self-harm presentations at Edinburgh, the year effect heterogeneity across areas (*I*^2^ 30.1%) was not statistically significant (Cochran’s *Q* test; *p* = 0.22) and the overall proportions were similar across the two years.

**Fig. 3:**
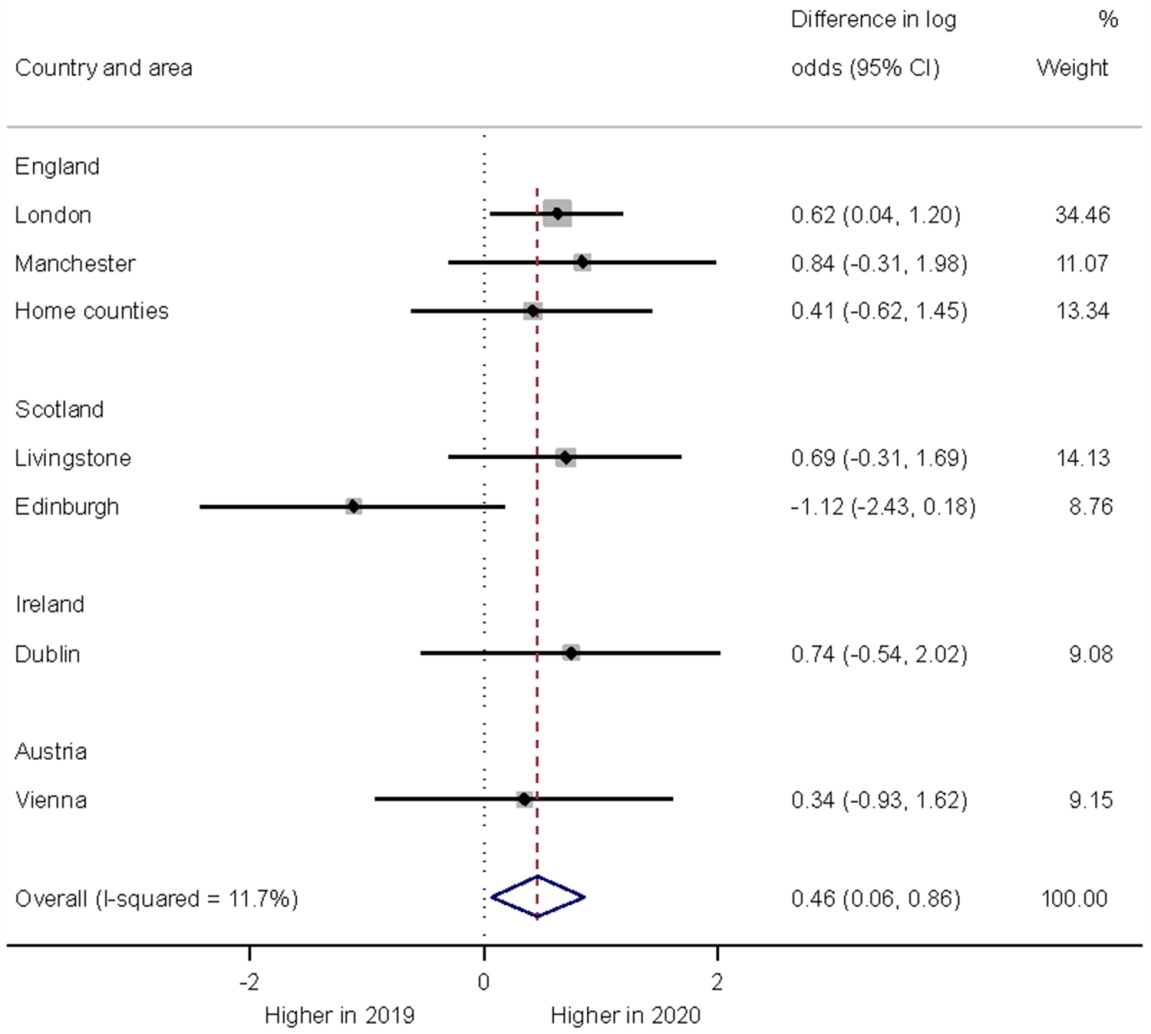
Forest plot illustrating year differences in the diagnosis of an emotional disorder in children and adolescents presenting with self-harm)

Out of the clinical diagnoses, emotional disorders were by far the most common. Emotional disorders also showed the largest observed increase in frequency in 2020 (from 58 to 66%). This increase tested statistically significant with an estimated odds ratio of 1.58, 95% confidence interval 1.06–2.36; *p* = 0.025. This finding was based on data from seven areas (Fig. 3). For some diagnoses, there was insufficient data to carry out a formal comparison, due to the diagnosis being rare. For other diagnoses, where there were sufficient data (behavioural disorders, neurodevelopmental disorders, substance use disorders, personality disorders), no statistically significant year differences were found.

The remaining clinical characteristics were not found to differ significantly between years (Table 2): the percentages of children and adolescents using a violent method for self-harm were similar in 2019 and 2020. The proportion of children and adolescents self-harming with suicide intent was 49% in 2019 and 55% in 2020 across all areas. However, this apparent increase in 2020 did not reach the threshold required for significance (*p* = 0.057). There were observed increases in the percentages of children and adolescents where a row with a family member or isolation was a precipitant of the presentation. However, neither of these tested significant at the 5% level. Finally, there was insufficient information to assess self-harm communications via social media, and there was no significant year difference in the percentages of self-harm presentations that involved alcohol use. There were observed increases in various distal risk factors in 2020, however, none of these year differences tested statistically significant.

### Clinical management

**Fig. 4:**
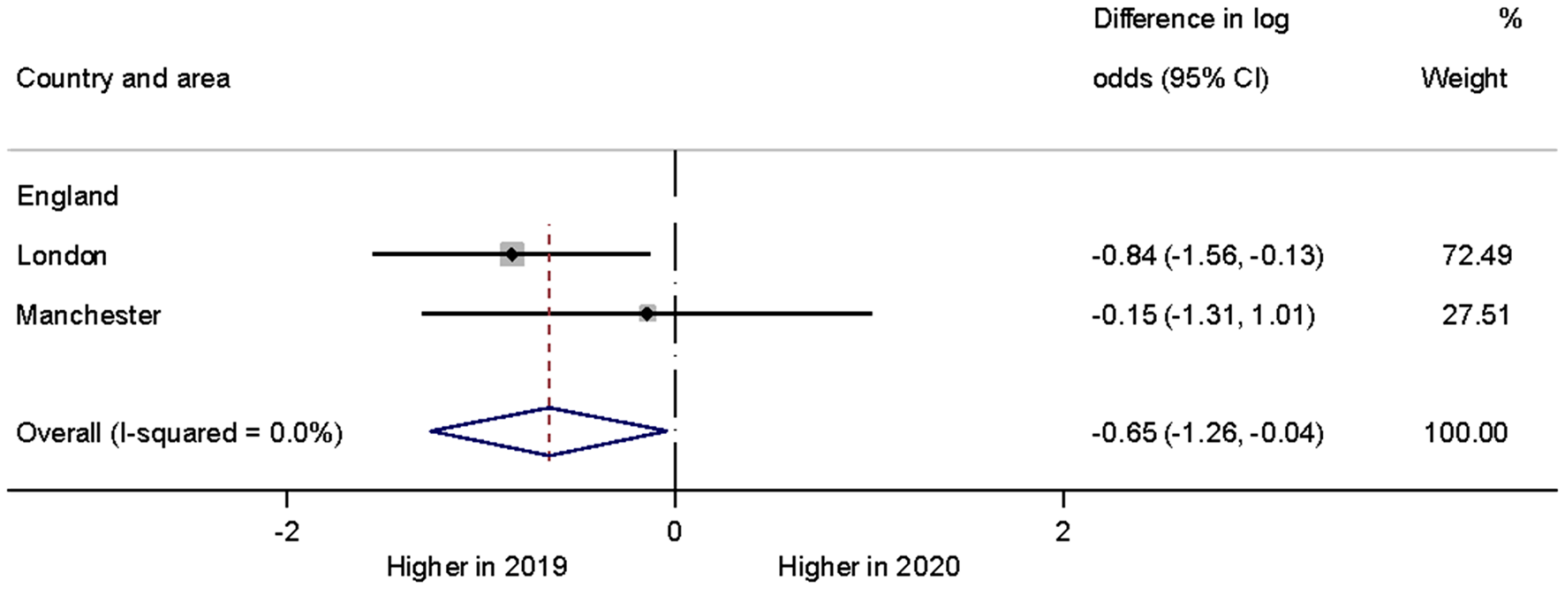
Forest plot illustrating year differences in admissions to an observation ward of children and adolescents with self-harm)

The proportion of children and adolescents who were admitted to an observation ward was significantly reduced in 2020 based on the two sites which had sufficient data for formal analyses (Fig. 4). There were no statistically significant year differences in other variables describing the young person’s hospital emergency contact.

Regarding the 2020 data, we asked researchers to consider if, in their judgement, the hospital presentation with self-harm was in any way related to covid-19. Researchers considered that 22.3% of 709 presentations from 14 areas were related to covid-19.

## Discussion

In this retrospective international cohort study we have found a decreased total number of presentations to hospital emergency departments during the Covid-19 lockdown, an increased proportion of children and adolescents with self-harm presentations and, contrary to our hypothesis, no increase in the proportion of children and adolescents with severe self-harm within those presenting with self-harm. The proportion of children and adolescents with previous hospital presentations for self-harm has increased, suggesting that young people with existing mental health problems were disproportionately affected during the lockdown, possibly due to a disruption of their established support networks. In further exploratory analyses, we found that in 2020 those presenting with self-harm were less likely to be admitted to an observation ward. The latter finding is surprising and may reflect an overall reluctance to keep patients in hospital for longer than was absolutely necessary.

### Could the reduction in the presentations be explained by an increase in inpatient psychiatric admissions?

**Fig. 5:**
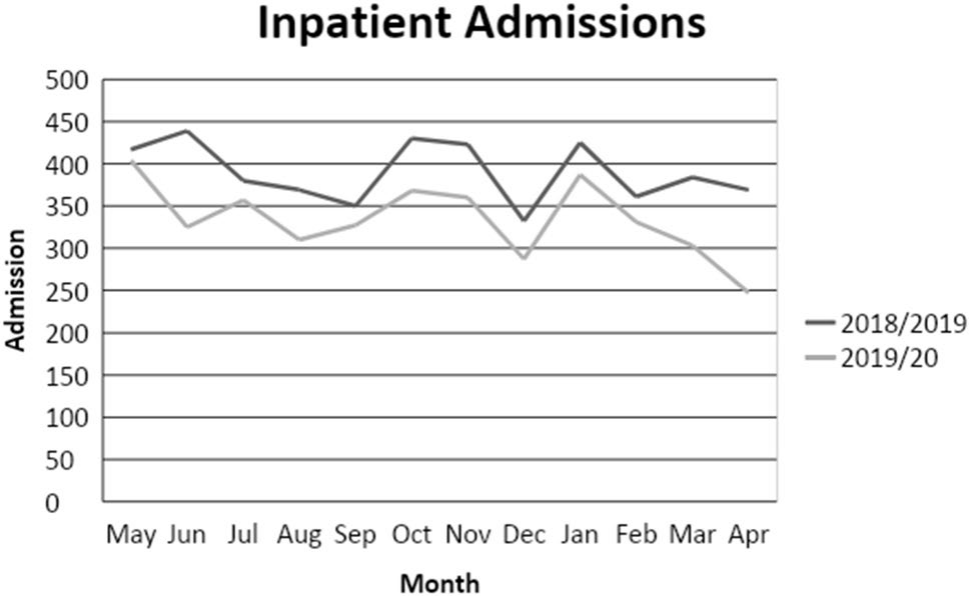
Monthly number of inpatient psychiatric admissions in England in 2018/19 and 2010/20**)**

The overall significant reduction in the number of presentations was not compensated for by an increase in inpatient psychiatric admissions, at least not in England. We used the National Commissioning Data Repository, an England-wide dashboard providing the most up-to-date data on a number of health services, including child and adolescent mental health services. NCDR contains information on temporal changes in inpatient psychiatric admissions of children and adolescents. We also used the data available from NHS Digital which provided data on total hospital emergency presentations in March and April 2020 compared with March and April 2019 in the UK. The total number of psychiatric inpatient admissions for children and adolescents in England in March 2020 was 303 (384 in 2019, 359 in 2018 and 414 in 2017 and 2016), the lowest number of admissions in any month since records began. In April 2020, this number further decreased to 247 (369 in 2019, 384 in 2018, 419 in 2017 and 388 in 2016) **(**Fig. 5).

It is, therefore, likely that a number of young people with severe psychiatric disorders have not received the treatment they required during the lockdown. Recently there has been growing evidence of an increase in the number of suicides in young people, which might indirectly support this suggestion [23]. The key learning point from this research should be noted by service planners: during any future lockdowns, intensive community care services with outreach capabilities should be prioritised, rather than boosting emergency hospital departments, which has occurred during the current lockdown. Other authors have reached similar conclusions, advocating for urgent re-thinking of the way services should be delivered [24].

### Sociodemographic characteristics and follow-up

We have found several important negative findings. We have found no evidence that the proportion of children and adolescents from ethnic minorities or deprived areas presenting to emergency departments with self-harm has changed during the pandemic. We have also found no differences in the offers of community follow-up appointments or the chances of young people attending these appointments. As the risk of suicide is greatest in the week after a hospital presentation with self-harm, this is an important finding. We have, however, measured any form of follow-up appointments, including phone calls and online appointments. The quality of these forms of follow-up has not been established. The total number of emergency presentations in England has reduced very substantially during the covid-19 lockdown. Using the NHS Digital database, the total number of hospital emergency presentations for all causes in England in April 2020 was 917,000, a decrease of 56.6% on the same month last year. These are the lowest number of attendances reported since this collection began and are likely to be a result of the covid-19 response. Our data, although indicative of a significant reduction in hospital presentations, is indicative of a smaller reduction in psychiatric emergency presentations.

### Incidence of self-harm

We report a decreased number of overall psychiatric presentations but also an increased proportion of children and adolescents with self-harm. Numerically, however, monthly incidence of children and adolescents presenting with self-harm to emergency departments during the covid-19 pandemic was lower than the year before. This is in contrast with previous hospital-based studies and primary care-based studies that have all indicated a continuous increase in these presentations pre-Covid-19 [4].

### Potential causes of the changes observed

The lower incidence of hospital presentations among this age group is potentially due to the quarantine measures. All included countries implemented school closures, international, and domestic travel restrictions by mid-March. Of importance, many children and adolescents stopped going to school in March and April 2020 and the pressure to perform during the examination periods was reduced. In addition, many children and adolescents are likely to have stayed at home more often, interacting with their parents and feeling part of a family. It may be that a proportion of those who would ordinarily have presented to hospital in crisis were able to find alternative coping strategies within the altered environment of lockdown. Also, many children and adolescents may have had fewer opportunities to engage in face-to-face encounters associated with increased risk of self-harm and psychiatric disorders, such as face-to-face bullying, using drugs and alcohol and engaging in risky behaviour. Although some evidence indicates that common mental disorders, especially emotional disorders are becoming more common during the periods of epidemics and that some children and adolescents might be at greater risk of violence and abuse at home, the numbers affected might be relatively low. We did find an increased proportion of children and adolescents with emotional disorders, however.

It is also possible that some young people chose not to attend emergency departments for fear of getting infected or spreading the infection. Carers’ and young people’s worries about contracting and spreading the virus might have serious adverse impact on young people’s health and well-being and may impact on health service use [25, 26]. On the other hand, increased risks of domestic violence and deteriorating parental mental health, which particularly affect young people who have already experienced Adverse Childhood Experiences (ACEs), have been reported during the lockdown [27] and, coupled with our findings, point of a number of young people not receiving services who might desperately need them.

Exposure to digital media and its potential impact on children and adolescents’ mental health is the centre of continued media debate. Such technologies can be helpful and facilitate access to care and support but there is also a suggestion that extreme “connectedness” could have detrimental effects, especially during the lockdown.

### Strengths and limitations of this study

Although we utilised a number of sources, including national (the NCDR and the NHS Digital in the discussion section) and local, the study did have some limitations. We described the characteristics of self-harm from an emergency department perspective. This was not a complete community sample of children and adolescents who harmed themselves. An illustration of this limitation is that self-cutting was less often recorded in our study and self-poisoning episodes predominated, whereas the opposite has been found in community surveys.

Despite including an international sample, more than half of the presentations were recorded in the UK. Whilst our meta-analyses were weighted, countries with small sample sizes were likely underrepresented.

The lower number of inpatient admissions may have been explained, at least in part, by the recent development of intensive community care services in the UK or temporary relief of academic stress, and not by the Covid-19 outbreak. Meanwhile, the comparatively low number of presentations in middle income countries could have been restricted by potentially lacking mental health care pathways or stigma to seek help.

Local electronic patient records also have limitations. They are not always completed comprehensively, and we did have some missing data. Different countries used different systems of patient records. Some participating countries at individual sites had to create electronic records for presentations to participate—this highlights the need for services to have electronic databases that can be interrogated—to better monitor changes over time and support advocacy and quality of care.

## Conclusion

This large study provides a unique hospital care perspective on self-harm and inpatient admissions among children and adolescents from 23 different hospitals and ten different countries. We found a noticeable decrease in recorded emergency presentations but no substantial increase in the proportion of children and adolescents with severe self-harm. This marked apparent decrease prompts the need to identify the causes of this phenomenon. Further development of appropriate interventions is needed as there is little evidence of a consistent clinical management approach for self-harm among children and adolescents, especially in hospital emergency departments. It is not clear how long this decrease is likely to last and if a compensatory increase following the quarantine will ensue. We also found a substantial decrease in inpatient psychiatric admissions. The findings have major implications for service planning if there is a second wave of Covid-19 or a future pandemic or a lockdown for any other public health emergency. Clinicians should prepare for more virtual and phone-based contacts with patients. Specific services for LAC should be supported by health systems as a matter of urgency where these do not exist. Services should re-configure in such a way that emergency department hospital staff are prepared for lower numbers of presentations and redeployment of staff from hospital-based care services to intensive community care services with outreach capabilities should be prioritised.

## Supplementary Information

Below is the link to the electronic supplementary material.

Supplementary file1 (PDF 596 KB)
