# Supplementary material for: Pandemic-related emergency psychiatric presentations for self-harm of children and adolescents in 10 countries (PREP-kids): a retrospective international cohort study
Source: Eur Child Adolesc Psychiatry. 2021 Mar 7;31(7):1–13. doi: 10.1007/s00787-021-01741-6 (PMC7937052; doi:10.1007/s00787-021-01741-6)

Supplementary materials

Table 1 Proportion of self-harm presentations out of all hospital emergency presentations (data available for 1,219 presentation in 2019 and 826 in 2020)

Area	2019	2020	Total
London	0·53	0·61	0·56
Manchester	0·80	0·96	0·86
Home counties	0·57	0·65	0·60
Livingstone	0·58	0·55	0·57
Edinburgh	0·72	0·75	0·73
Dublin	0·54	0·64	0·59
Cagliari	0·36	0·37	0·36
Turin	0·67	0·62	0·64
Vienna	0·20	0·28	0·23
Budapest	0·29	0·44	0·33
Belgrade	0·55	1·00	0·67
Istanbul	0·33	0·60	0·45
Muscat	0·73	0·67	0·70
Dubai	0·54	0·50	0·53
Total	0·50	0·57	0·53

Table 2 Proportion of severe self-harm presentations out of all self-harm presentations (data available for 605 presentation in 2019 and 649 in 2020)

Area	2019	2020	Total
London	0·15	0·18	0·16
Manchester	0·31	0·30	0·30
Home counties	0·19	0·15	0·17
Livingstone	0·33	0·13	0·26
Edinburgh	0·16	0·39	0·24
Dublin	0·08	0·15	0·11
Cagliari	0·32	0·50	0·38
Turin	0·11	0·25	0·18
Vienna	0·00	0·04	0·02
Budapest	0·00	0·25	0·10
Belgrade	0·75	0·63	0·70
Istanbul	0·50	0·17	0·30
Muscat	0·00	0·38	0·16
Dubai	0·29	0·50	0·33
Total	0·19	0·19	0·19

Table 3 Proportion of children and adolescents with emotional disorders out of all self-harm presentations (data available for 520 presentation in 2019 and 384 in 2020)

Area	2019	2020	Total
London	0·52	0·67	0·58
Manchester	0·46	0·67	0·56
Home counties	0·83	0·88	0·85
Livingstone	0·40	0·57	0·46
Edinburgh	0·38	0·17	0·30
Dublin	0·81	0·90	0·85
Cagliari	0·58	0·80	0·66
Turin	0·88	0·57	0·73
Vienna	0·40	0·49	0·45
Budapest	0·83	1·00	0·90
Belgrade	0·83	0·88	0·85
Istanbul	1·00	0·83	0·90
Muscat	0·27	0·25	0·26
Dubai	0·71	1·00	0·78
Total	0·58	0·66	0·61

Table 4 Proportion of children and adolescents admitted to observation wards out of all children and adolescents with self-harm presentations (data available for 548 presentation in 2019 and 422 in 2020)

**Area**	**2019**	**2020**	**Total**
London	0·34	0·18	0·27
Manchester	0·41	0·37	0·39
Home counties	0·04	0·03	0·04
Livingstone	0·02	0·03	0·03
Edinburgh	0·02	0·08	0·04
Dublin	0·00	0·00	0·00
Cagliari	0·26	0·00	0·17
Turin	0·10	0·38	0·22
Vienna			
Budapest	0·00	0·00	0·00
Belgrade	0·00	0·00	0·00
Istanbul	0·00	0·33	0·20
Muscat	0·00	0·00	0·00
Dubai	0·14	0·00	0·11
Total	0·13	0·09	0·11

Table 5 Characteristics of included hospitals

Hospital	City	Country	Observation ward (yes/no)	Multidisciplinary paediatric liaison team (yes/no)	Out-of-hours child/adolescent mental health assessment service (yes/no)	7-day follow-up after a presentation with self-harm required (yes/no)
Temple Street Children's University Hospital	Dublin	Republic of Ireland	no	yes	yes	no
Royal Manchester Children's Hospital	Manchester	United Kingdom	yes	yes	yes	yes
Dept. of Child and Adolescent Psychiatry, Medical University of Vienna	Vienna	Austria	yes	no	yes	no
Clinic for Neurology and Psychiatry for Children and Youth	Belgrade	Serbia	no	no	yes	no
University of Turin - Child & Adolescent Neuropsychiatry	Turin	Italy	yes	yes	yes	no
Children's Health Ireland at Crumlin	Dublin	Republic of Ireland	no	no	no	no
Vadaskert Alapitvany	Budapest	Hungary	no	no	yes	no
University of Cagliari - Child & Adolescent Neuropsychiatry Unit, AOB, Cagliari	Cagliari	Italy	yes	yes	yes	no
Acıbadem Mehmet Ali Aydınlar	Istanbul	Turkey	yes	yes	yes	yes

University Medical Faculty, Atakent Hospital						
CNWL NHS Foundation Trust - St Mary's Hospital, Chelsea and Westminster Hospital, Northwick Park Hospital and Hillingdon Hospital A&Es	London	United Kingdom	yes	yes	yes	yes
Sultan Qaboos University Hospital & Al-Massara Hospital	Muscat	Oman	no	no	yes	yes
Royal Edinburgh Hospital	Edinburgh	United Kingdom	no	no	yes	no
Royal Infirmary of Edinburgh	Edinburgh	United Kingdom	yes	yes	yes	no
Royal Hospital for Sick Children	Edinburgh	United Kingdom	no	no	yes	no
St John's Hospital	Livingston	United Kingdom	Yes	no	yes	no
Lister Hospital	Stevenage	United Kingdom	yes	yes	yes	yes
Watford General Hospital	Watford	United Kingdom	yes	yes	yes	yes
Berkshire Healthcare NHS Foundation Trust	Berkshire	United Kingdom	yes	no	yes	yes
Kings College Hospital, Paediatric Liaison Service, South London and Maudsley NHS	London	United Kingdom	yes	yes	yes	yes

Foundation Trust						
St Thomas Hospital, Paediatric Liaison Service, South London and Maudsley NHS Foundation Trust	London	United Kingdom	yes	yes	no	yes
Croydon University Hospital	London	United Kingdom	no	yes	yes	yes
Rashid Hospital, Dubai Hospital and Latifa hospital - Dubai Health Authority	Dubai	United Arab Emirates	yes	yes	yes	no
Lewisham University Hospital	London	United Kingdom	yes	yes	yes	yes

Figure 1 Forest plot illustrating year differences in the proportion of children and adolescents presenting with previous community self-harm in the entire sample

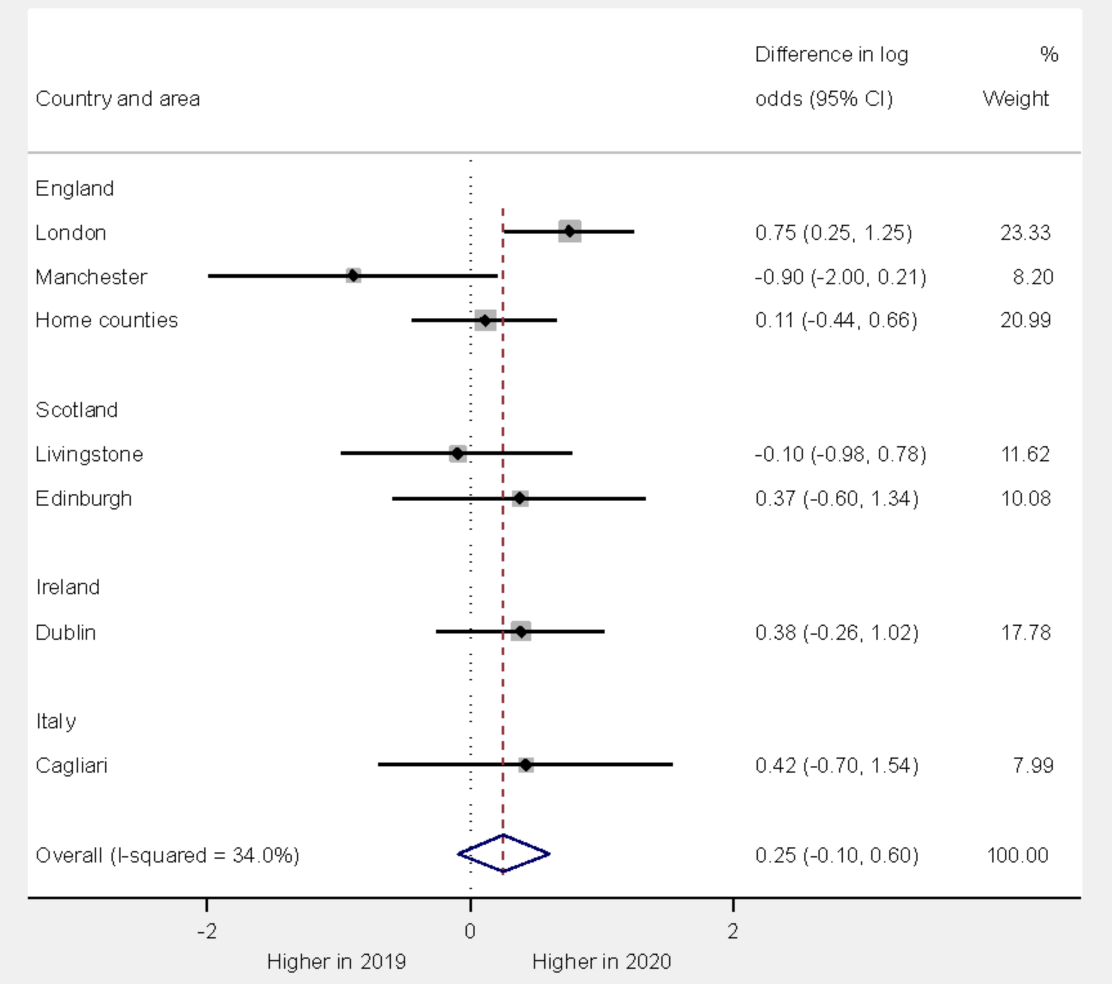

SOCIODEMOGRAPHIC CHARACTERISTICS OF CHILDREN AND ADOLESCENTS PRESENTING WITH SELF HARM

Figure 2 Forest plot illustrating year differences in the proportion of females in the sub-sample of children and adolescents presenting with self-harm

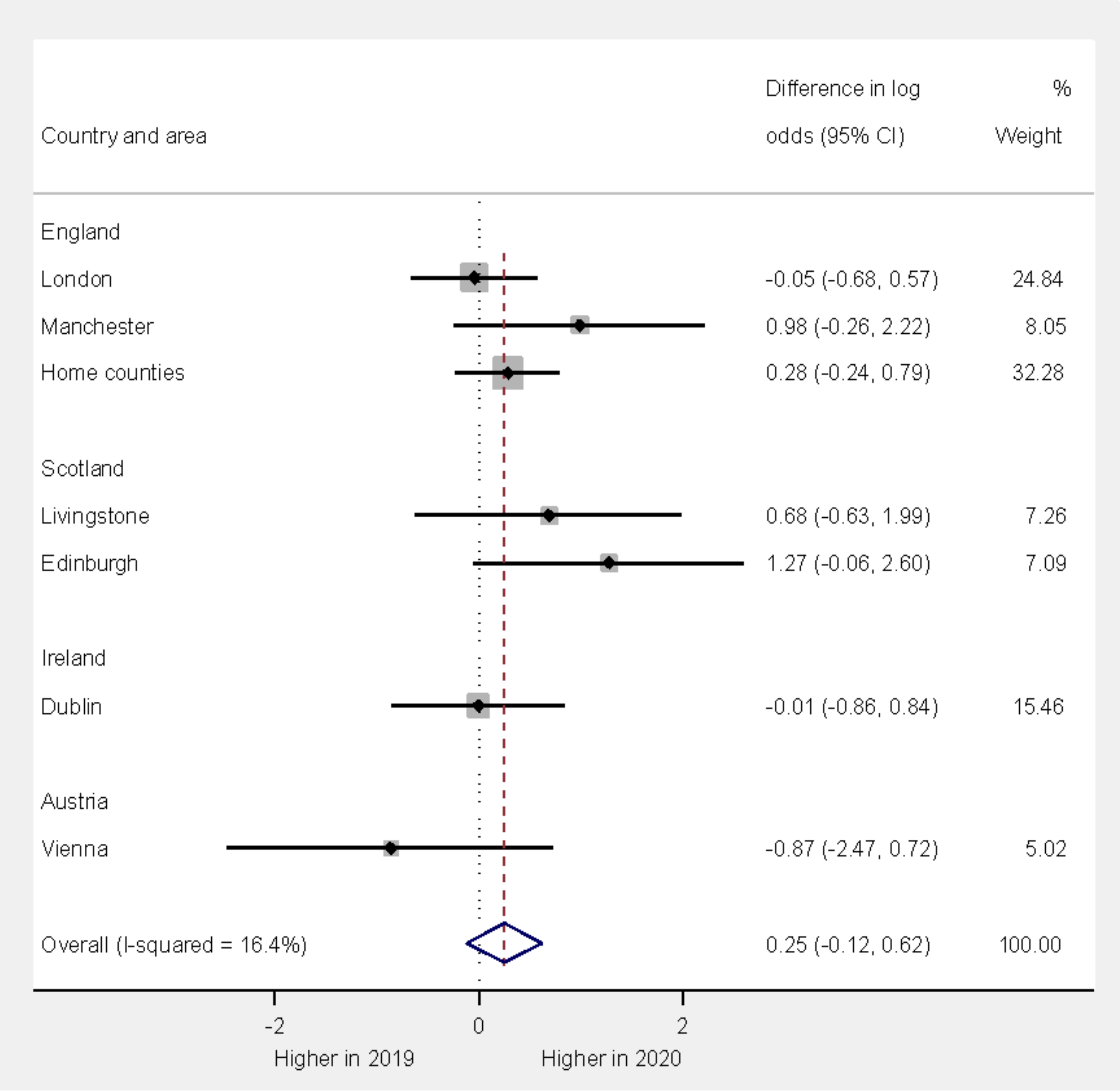

Figure 3 Forest plot illustrating year differences in age in children and adolescents presenting with self-harm

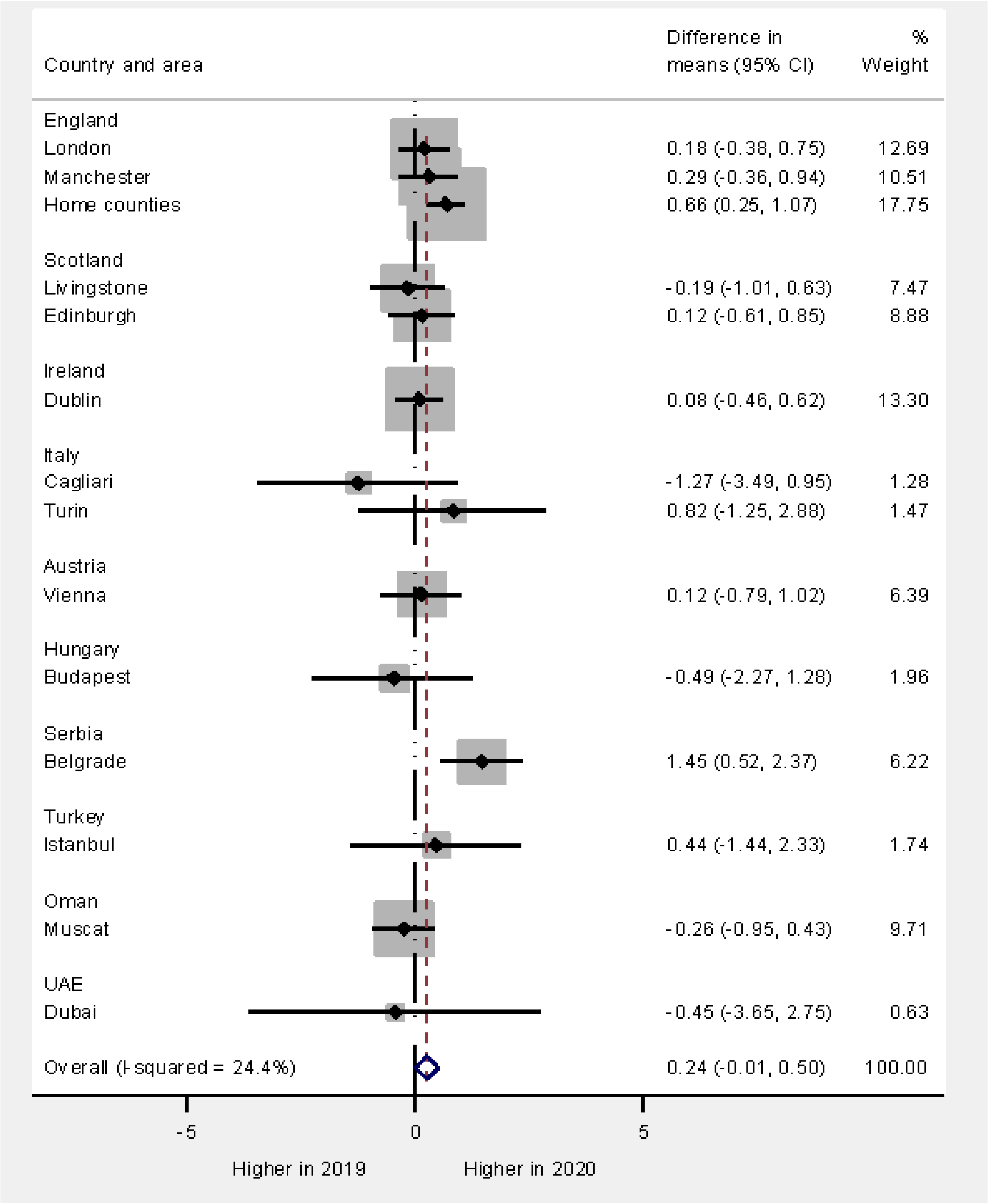

Figure 4 Forest plot illustrating year differences in the proportion of children and adolescents from the dominant ethnic group in children and adolescents presenting with self-harm

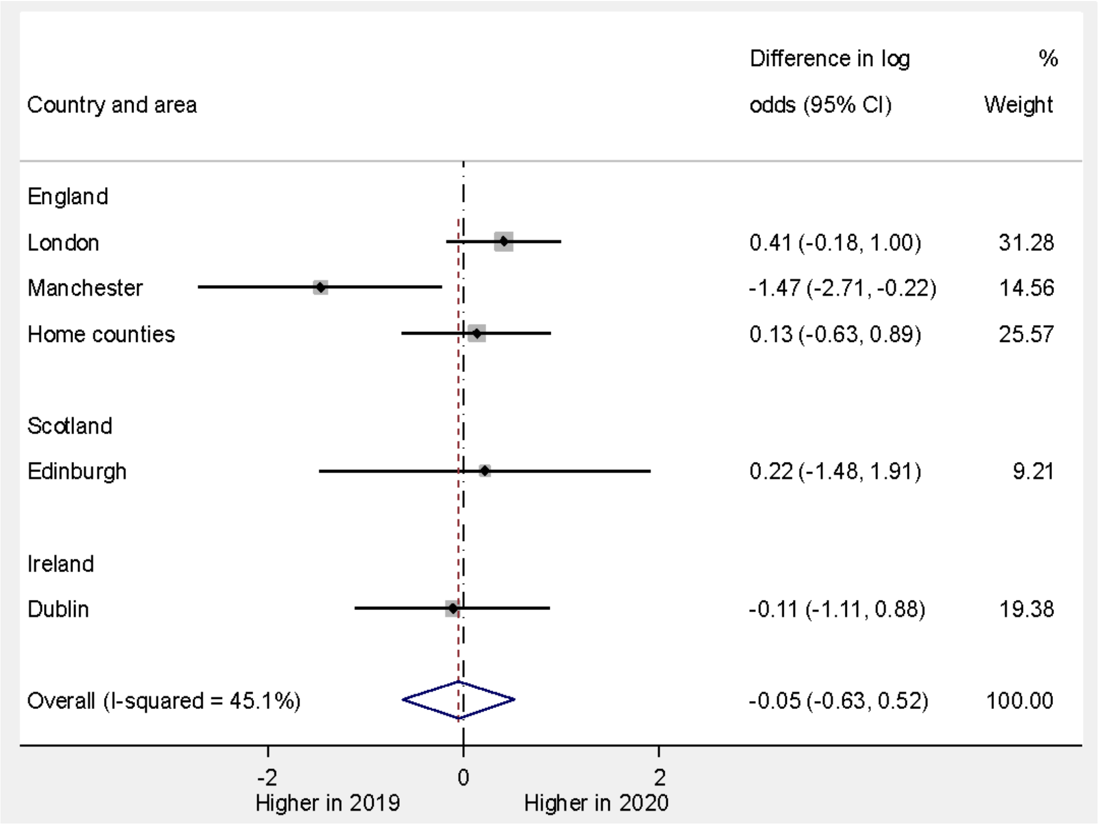

Figure 5 Forest plot illustrating year differences in the deprivation decile in children and adolescents presenting with self-harm

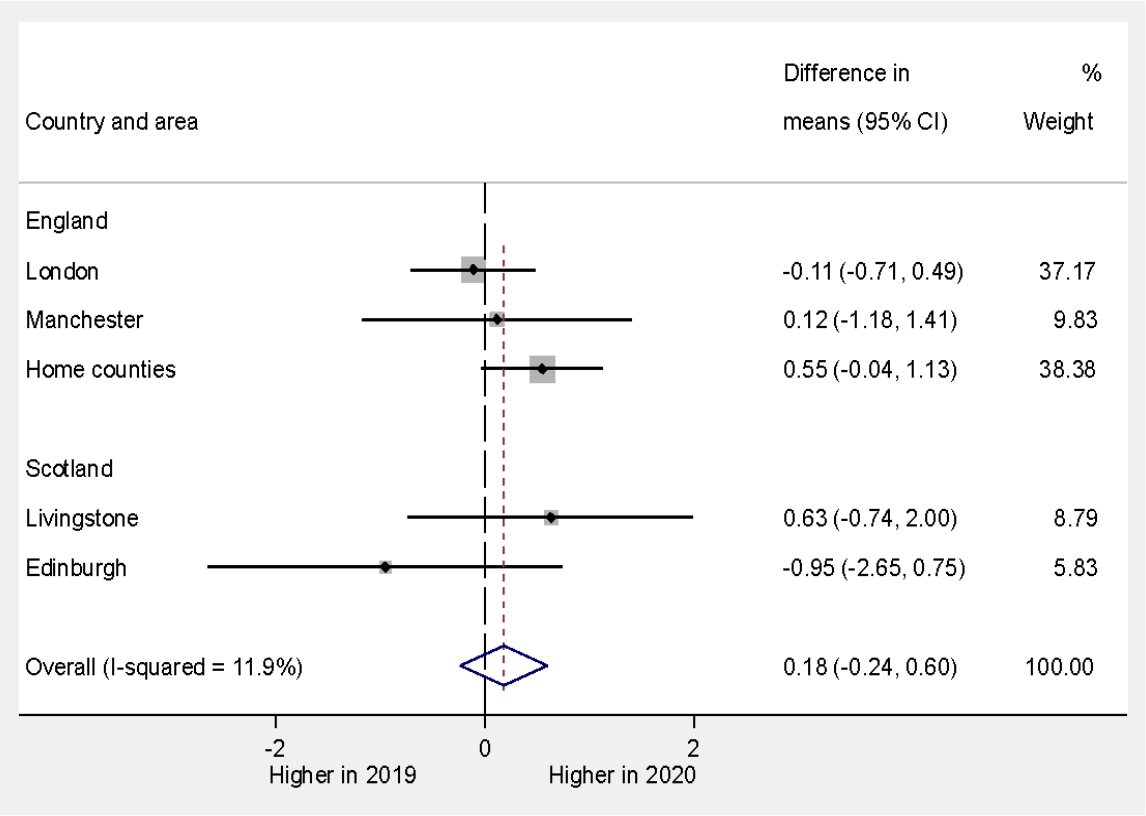

Figure 6 Forest plot illustrating year differences in the proportion of children and adolescents in Education, Employment or Training (EET) out of all children and adolescents presenting with self-harm

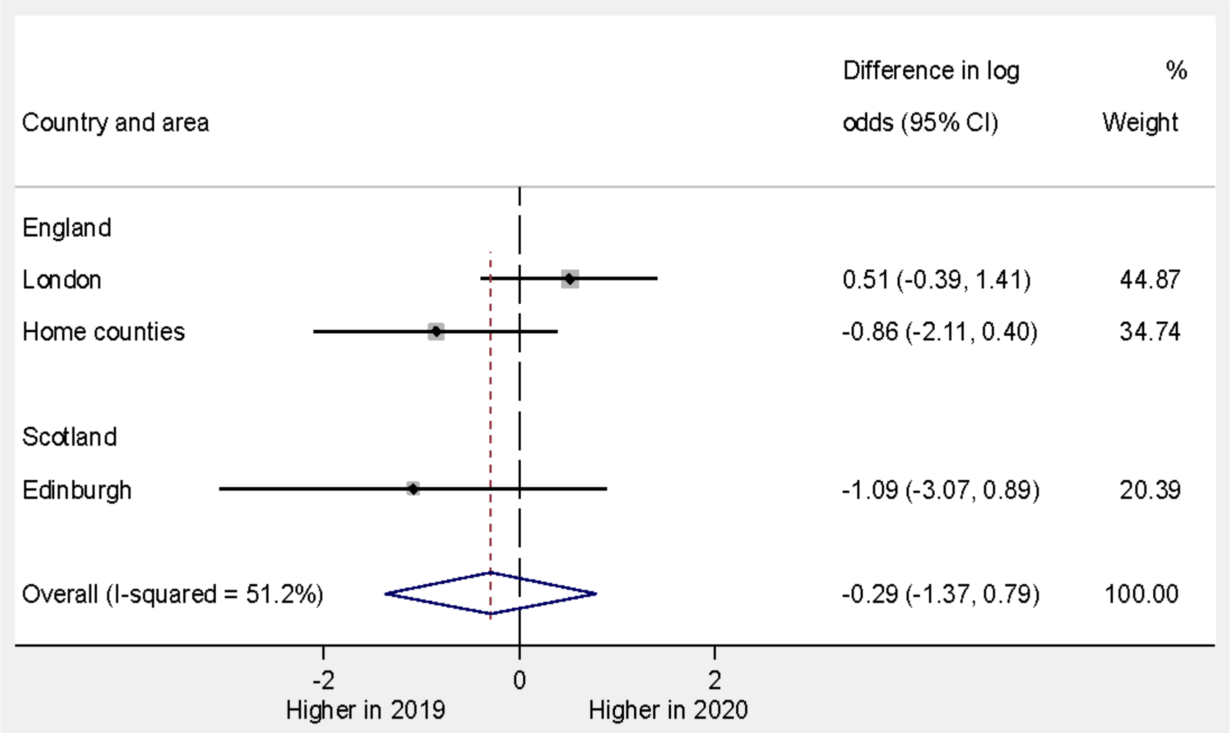

Figure 7 Forest plot illustrating year differences in the proportion young people looked after by local authority out of all children and adolescents presenting with self-harm

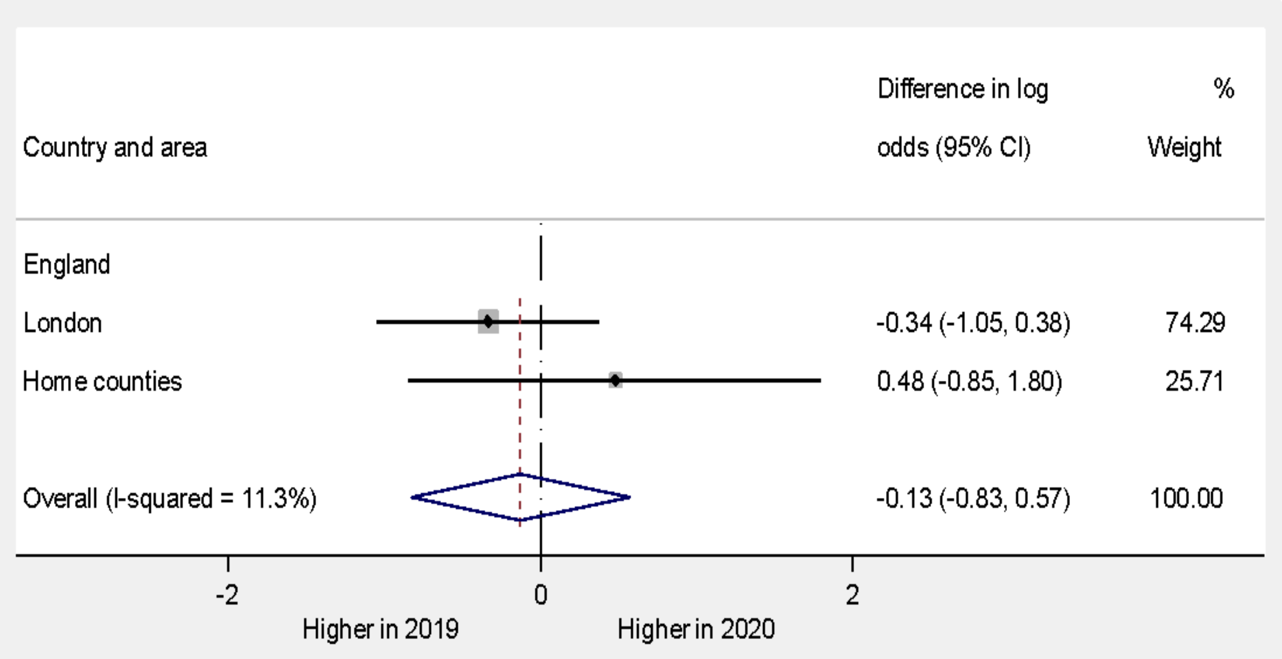

Figure 8 Forest plot illustrating year differences in the proportion of children and adolescents whose parents live together out of all children and adolescents not looked after by local authority presenting with self-harm

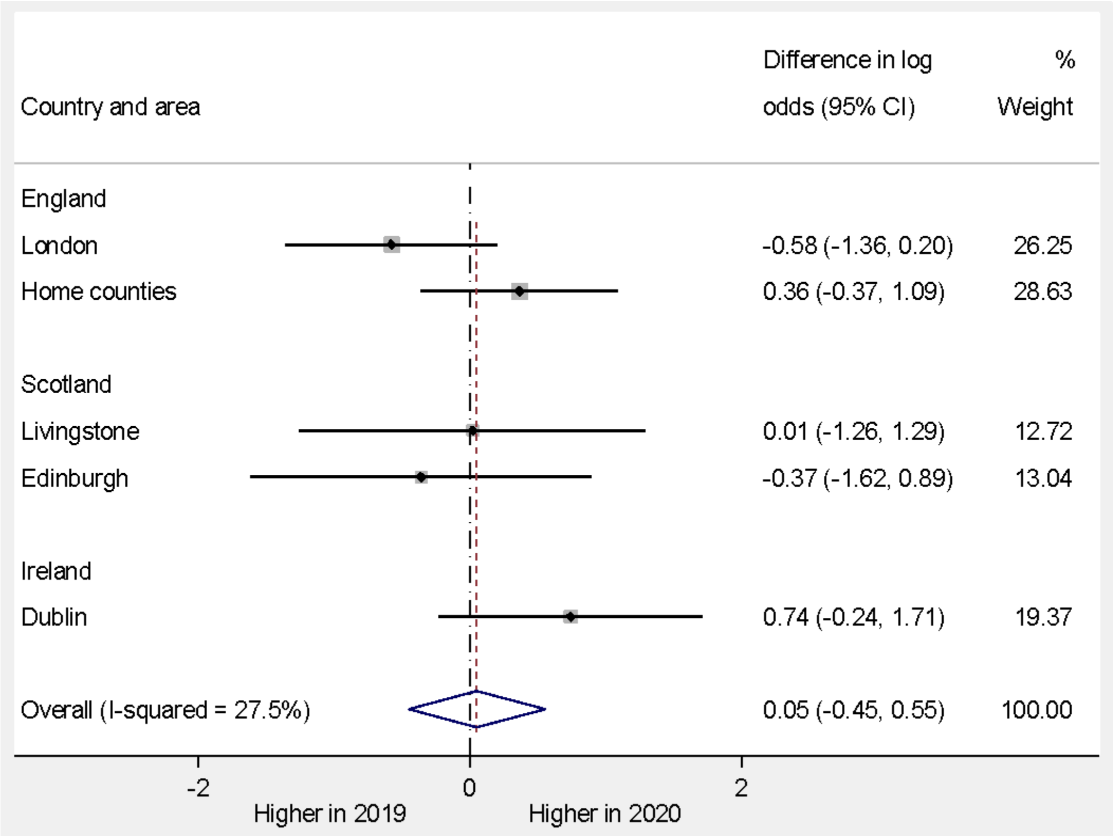

CLINICAL CHARACTERISTICS OF CHILDREN AND ADOLESCENTS PRESENTING WITH SELF HARM: DIAGNOSIS

Figure 9 Forest plot illustrating year differences in the diagnosis of a behavioural disorder in children and adolescents presenting with self-harm

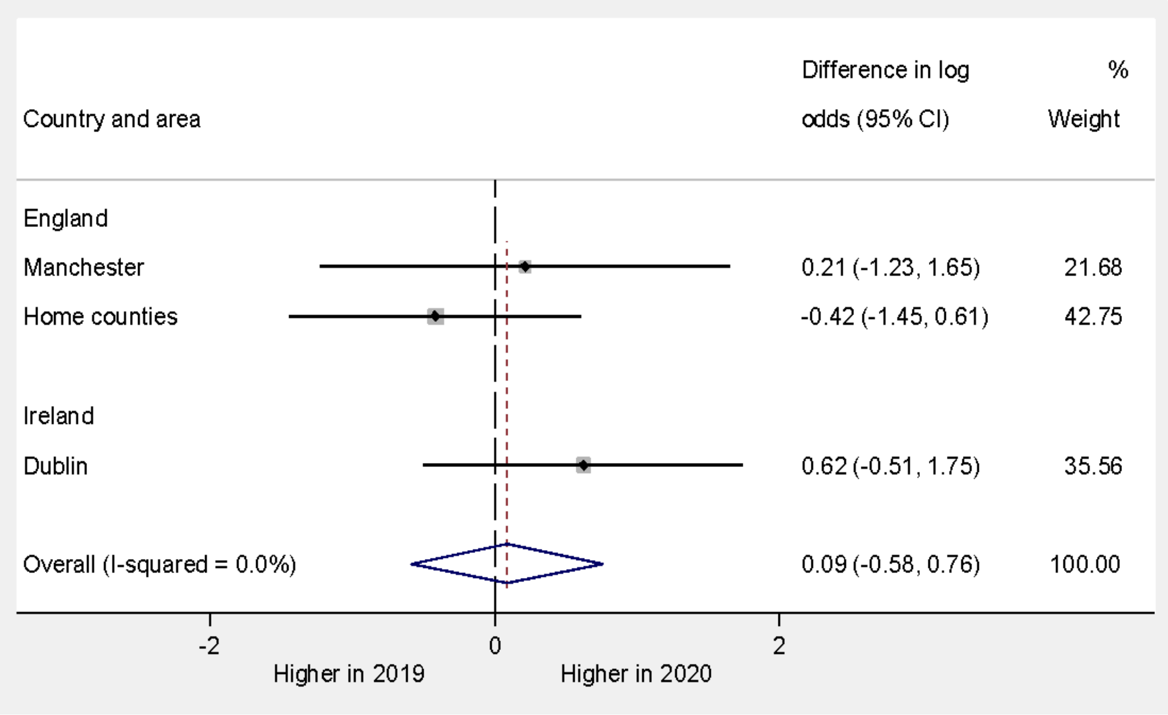

Figure 10 Forest plot illustrating year differences in the diagnosis of a neurodevelopmental disorder in children and adolescents presenting with self-harm

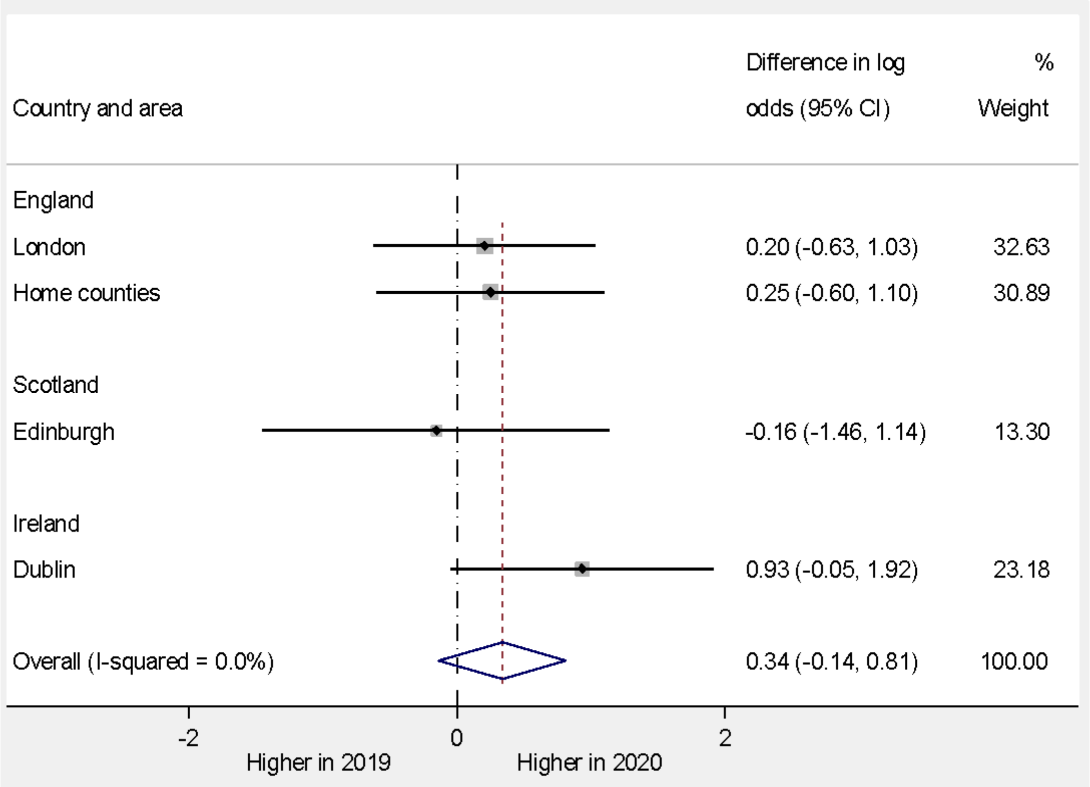

Figure 11 Forest plot illustrating year differences in the diagnosis of a substance misuse disorder in children and adolescents presenting with self-harm

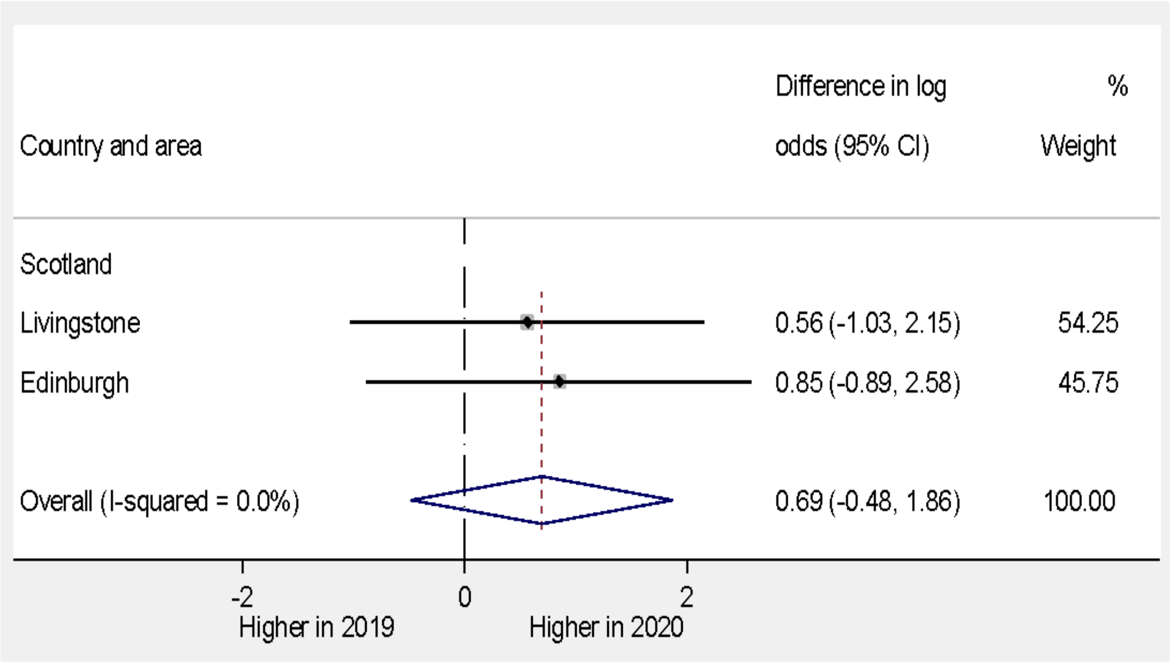

Figure 12 Forest plot illustrating year differences in the diagnosis of a personality disorder in children and adolescents presenting with self-harm

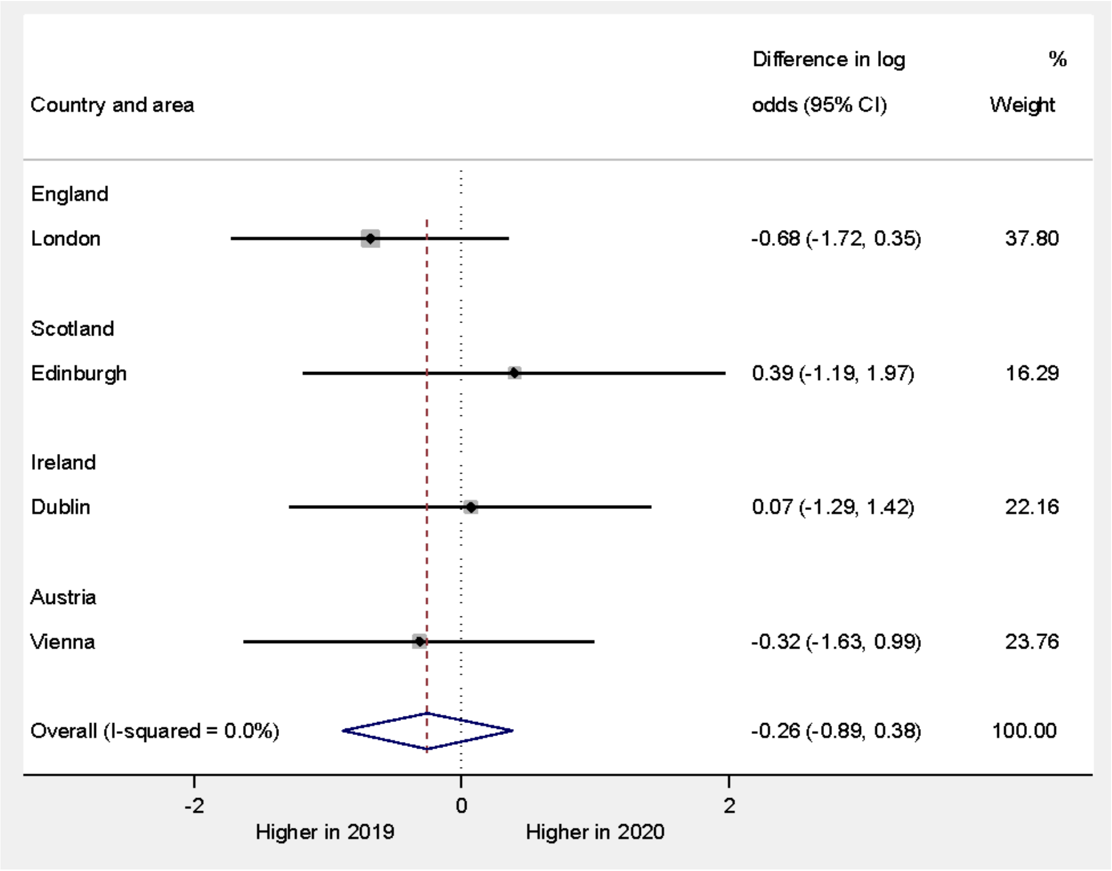

CLINICAL CHARACTERISTICS OF CHILDREN AND ADOLESCENTS PRESENTING WITH SELF HARM: POXIMAL RISK FACTORS

Figure 13 Forest plot illustrating year differences in the proportion of children and adolescents who used a violent method for self-harm out of all children and adolescents presenting with self-harm

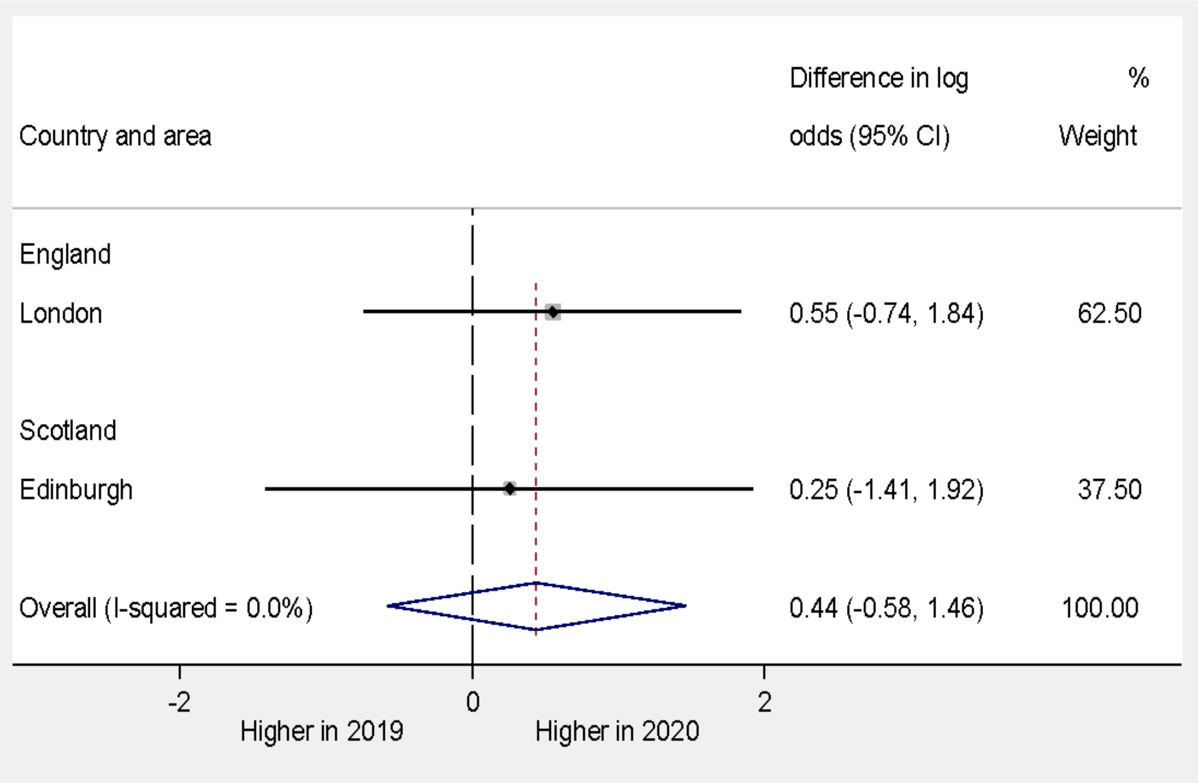

Figure 14 Forest plot illustrating year differences in the proportion of children and adolescents who expressed suicide intent out of all children and adolescents presenting with self-harm

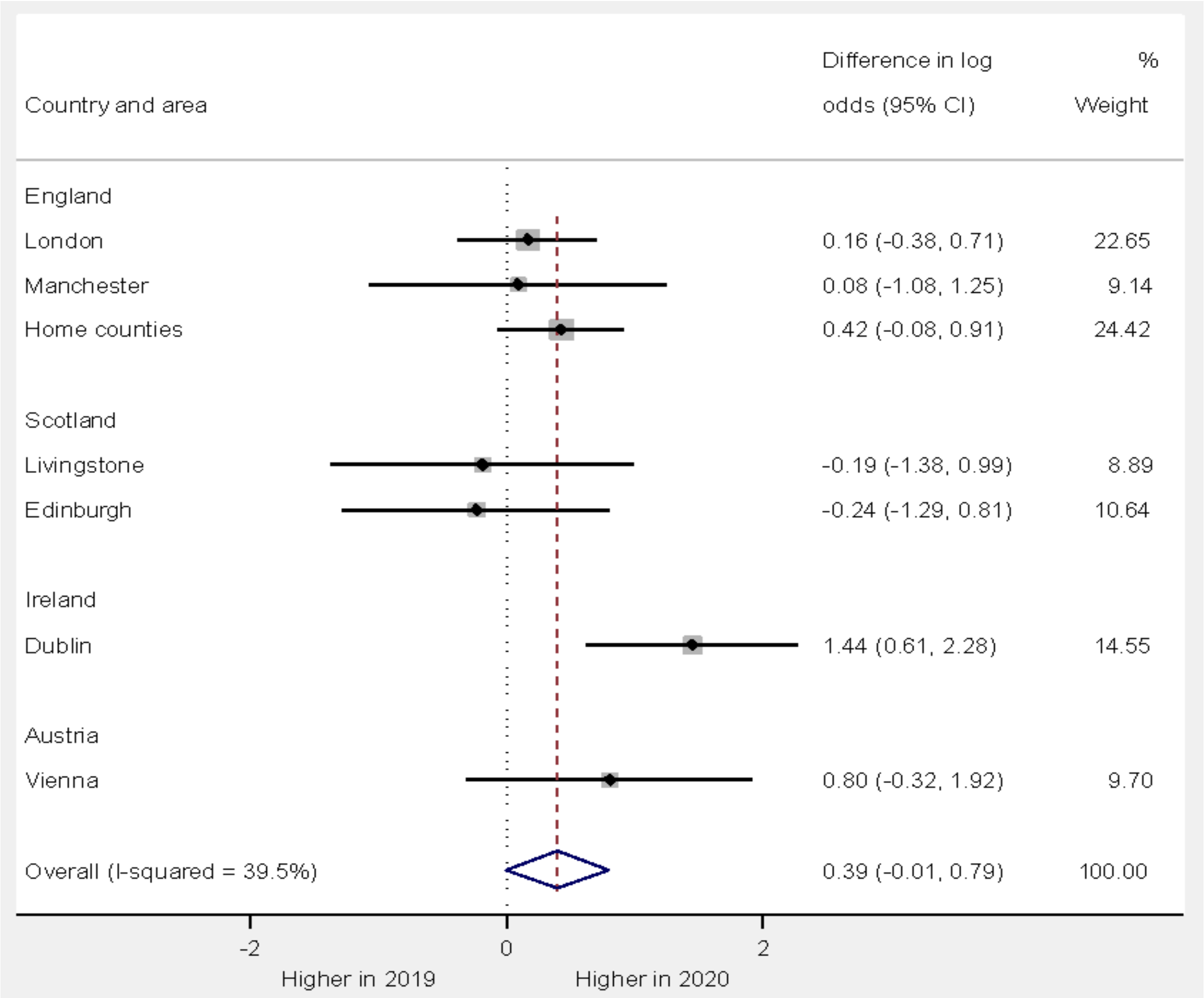

Figure 15 Forest plot illustrating year differences in the proportion of children and adolescents whose self-harm was precipitated by a row with a family member

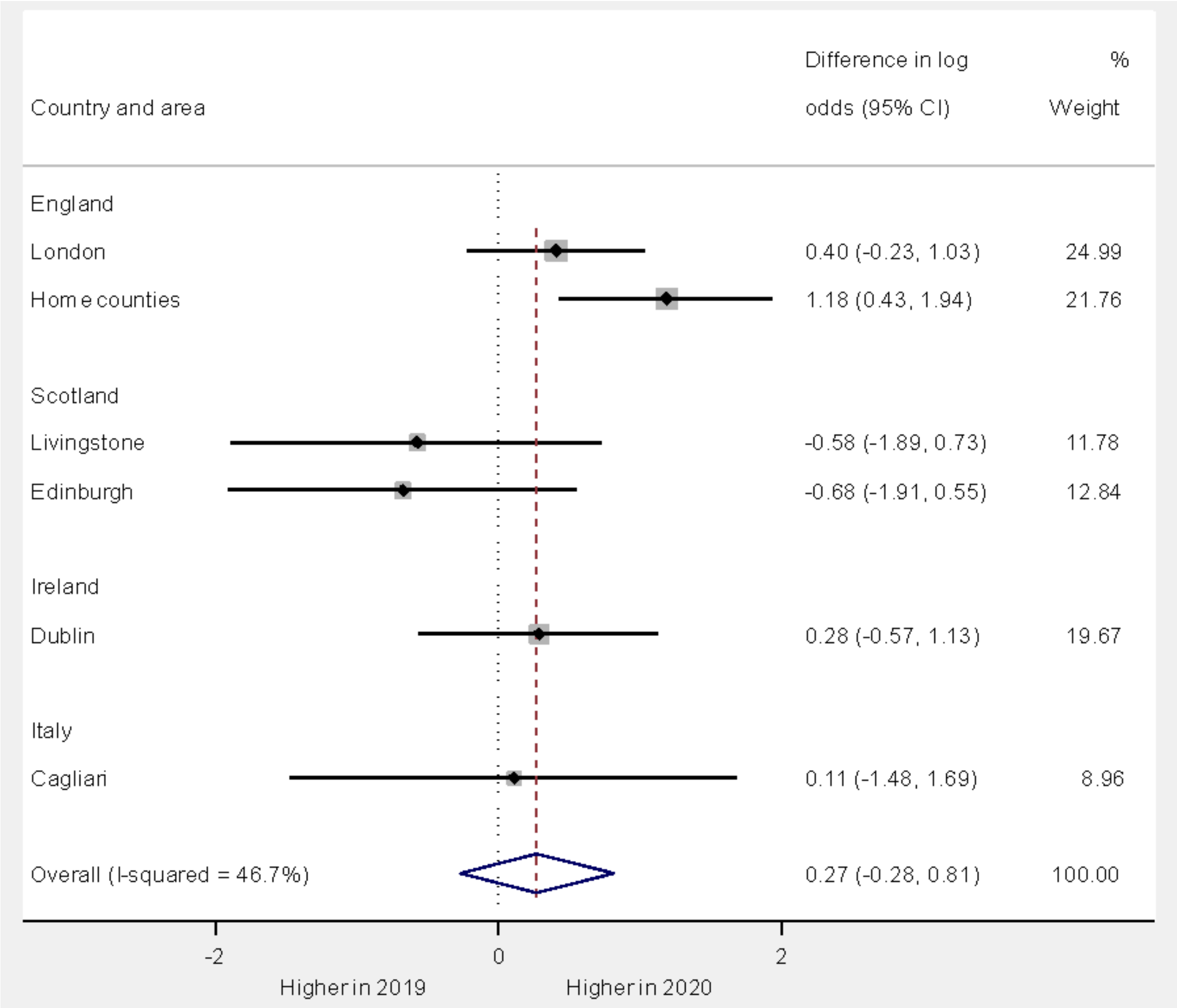

Figure 16 Forest plot illustrating year differences in the proportion of children and adolescents whose self-harm was precipitated social isolation

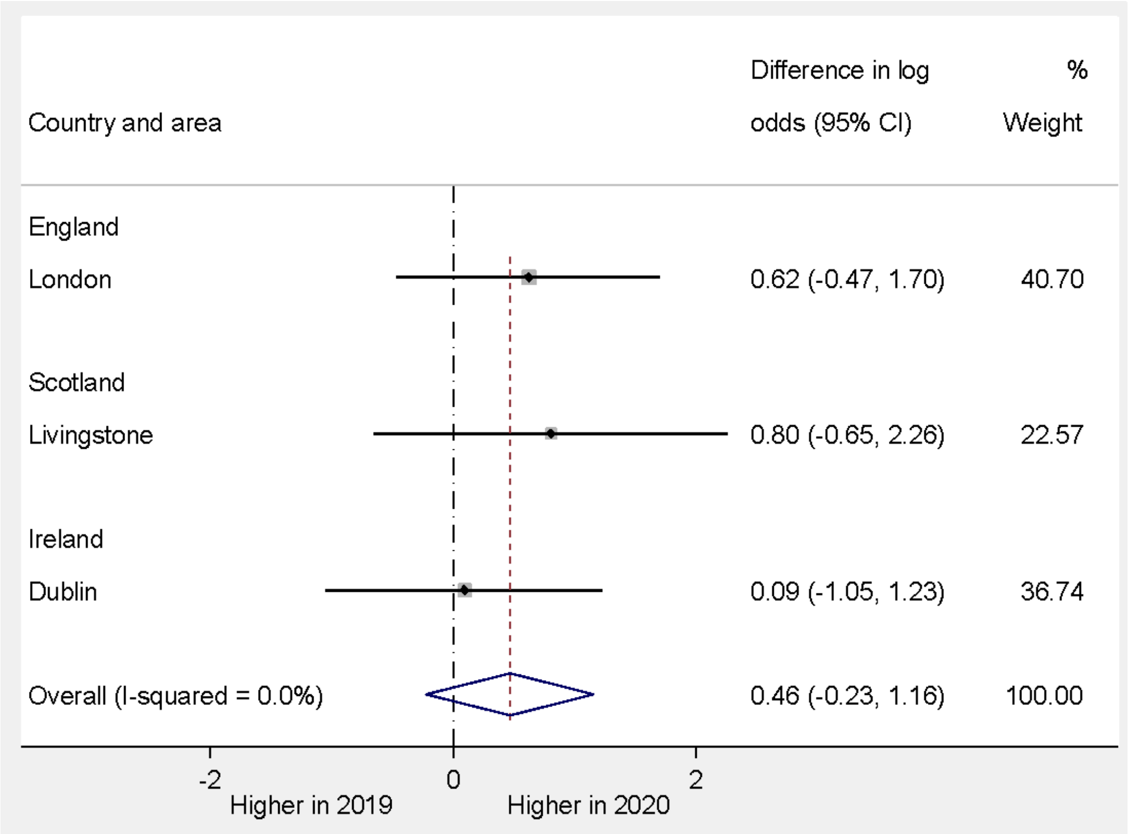

Figure 17 Forest plot illustrating year differences in the proportion of children and adolescents who ingested alcohol at the time immediately before, during or after self-harm

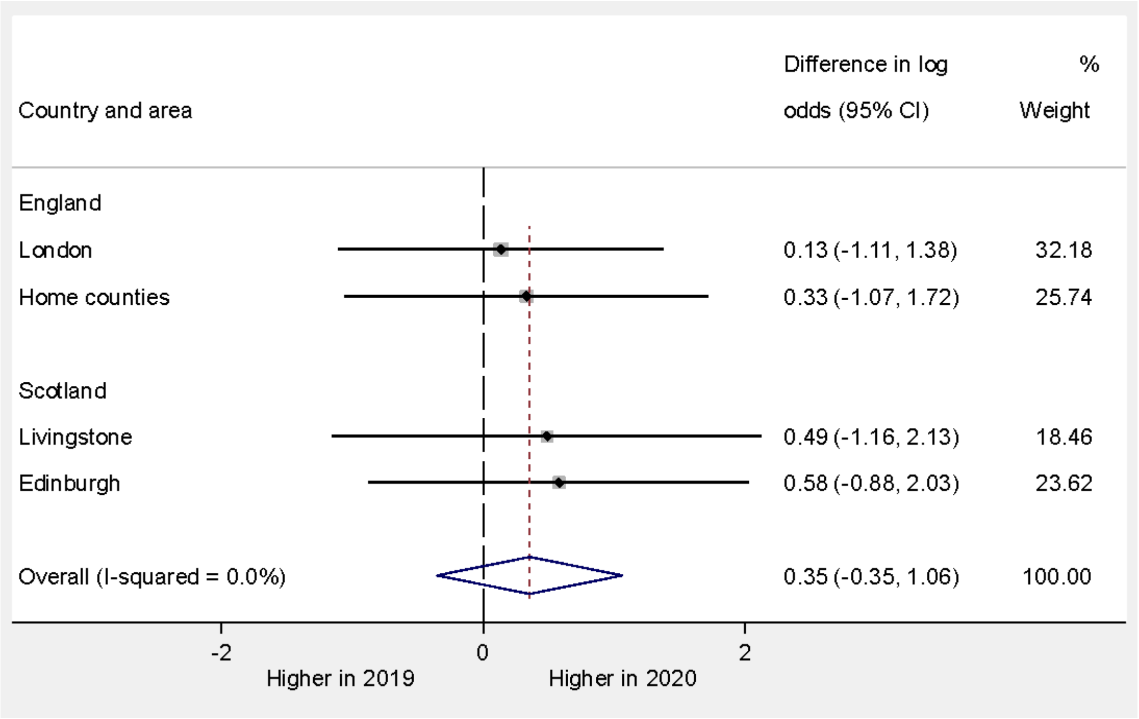

CLINICAL CHARACTERISTICS OF CHILDREN AND ADOLESCENTS PRESENTING WITH SELF HARM: DISTAL RISK FACTORS

Figure 18 Forest plot illustrating year differences in the proportion of children and adolescents with previous self-harm presentations to emergency hospital departments out of all children and adolescents presenting with self-harm

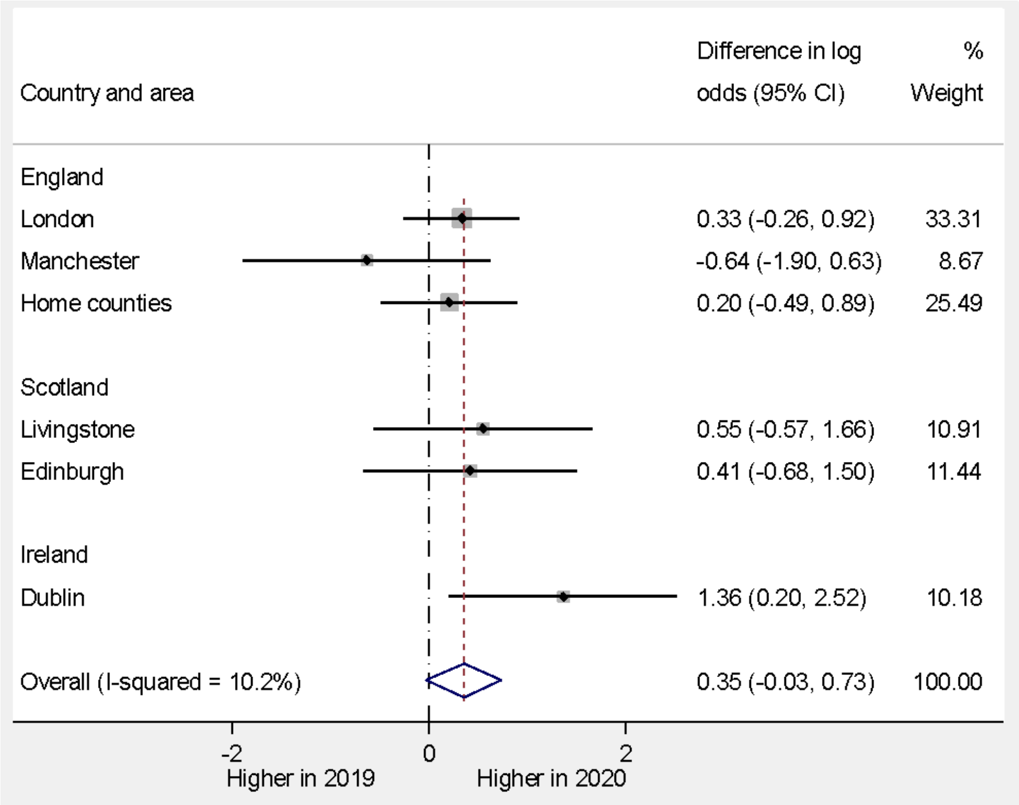

Figure 19 Forest plot illustrating year differences in the proportion of children and adolescents with previous presentations to emergency hospital departments for any reason other than self-harm, out of all children and adolescents presenting with self-harm

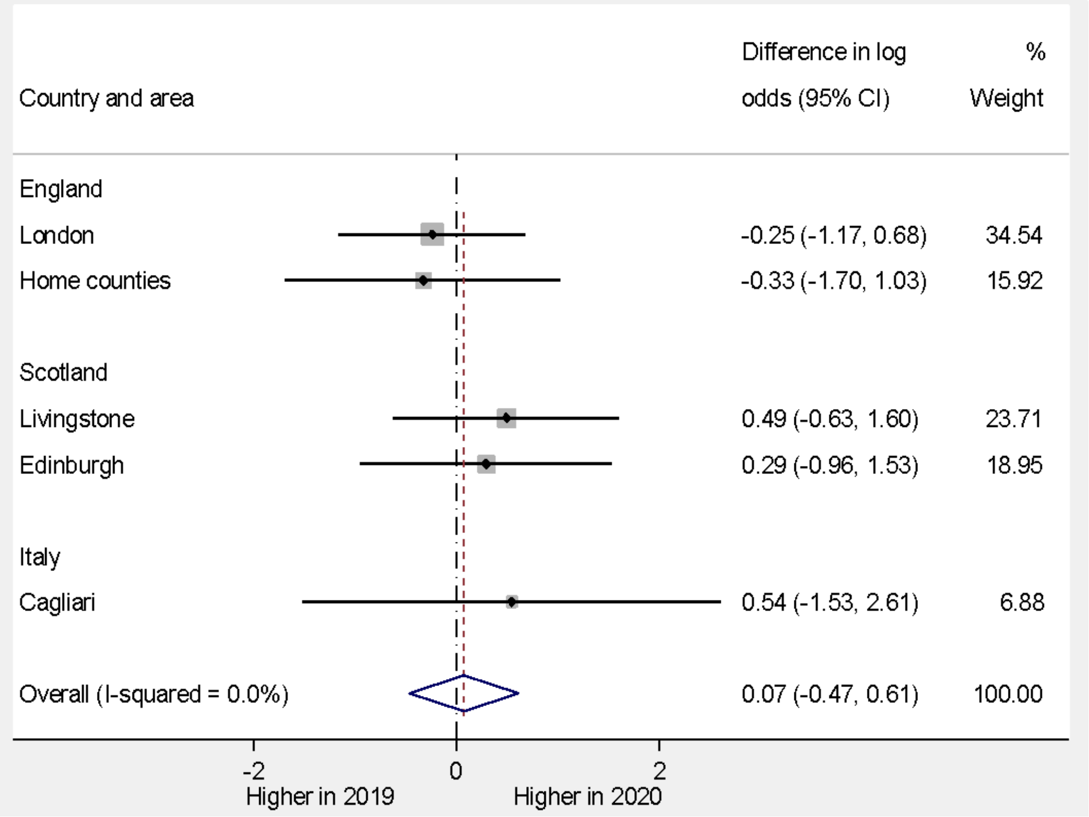

Figure 20 Forest plot illustrating year differences in the proportion of children and adolescents with previous community self-harm out of all children and adolescents presenting with self-harm

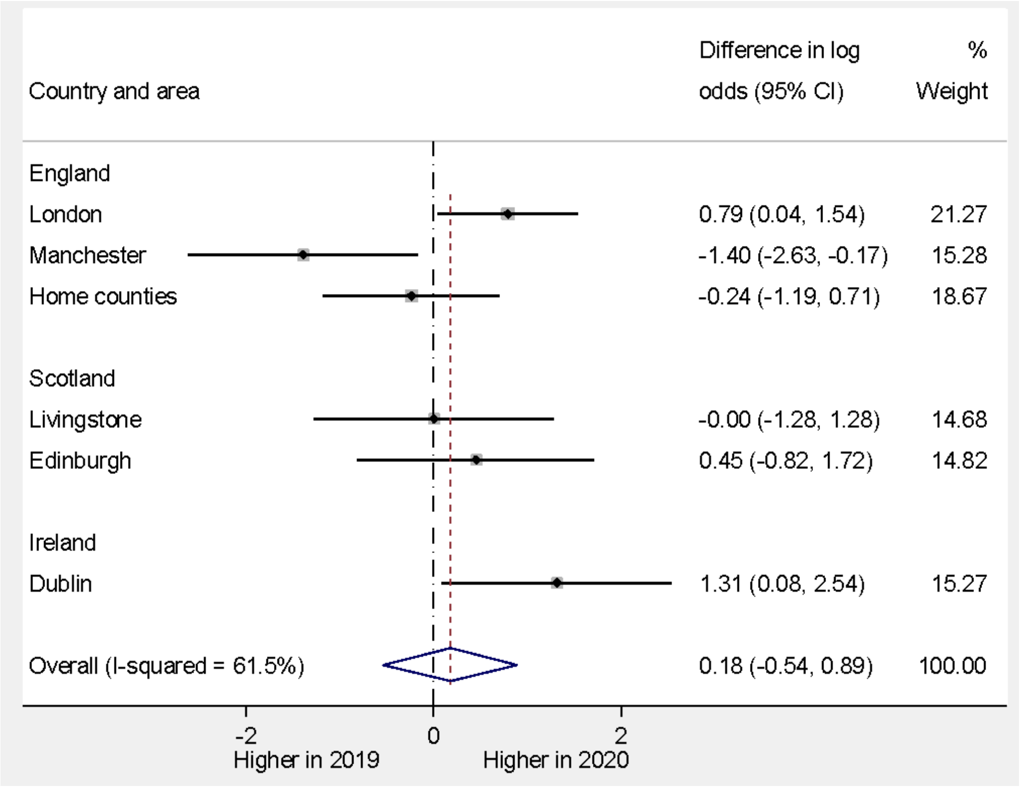

Figure 21 Forest plot illustrating year differences in the proportion of children and adolescents with previous psychiatric inpatient treatment out of all children and adolescents presenting with self-harm

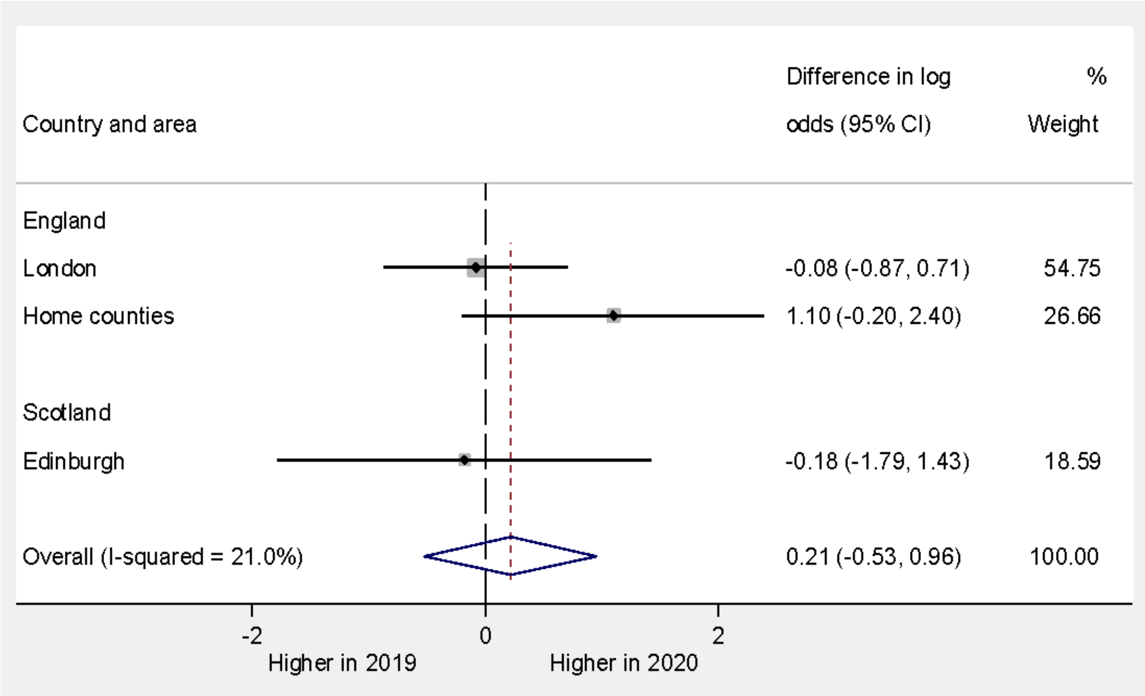

Figure 22 Forest plot illustrating year differences in the proportion of children and adolescents with a family history of self-harm out of all children and adolescents presenting with self-harm

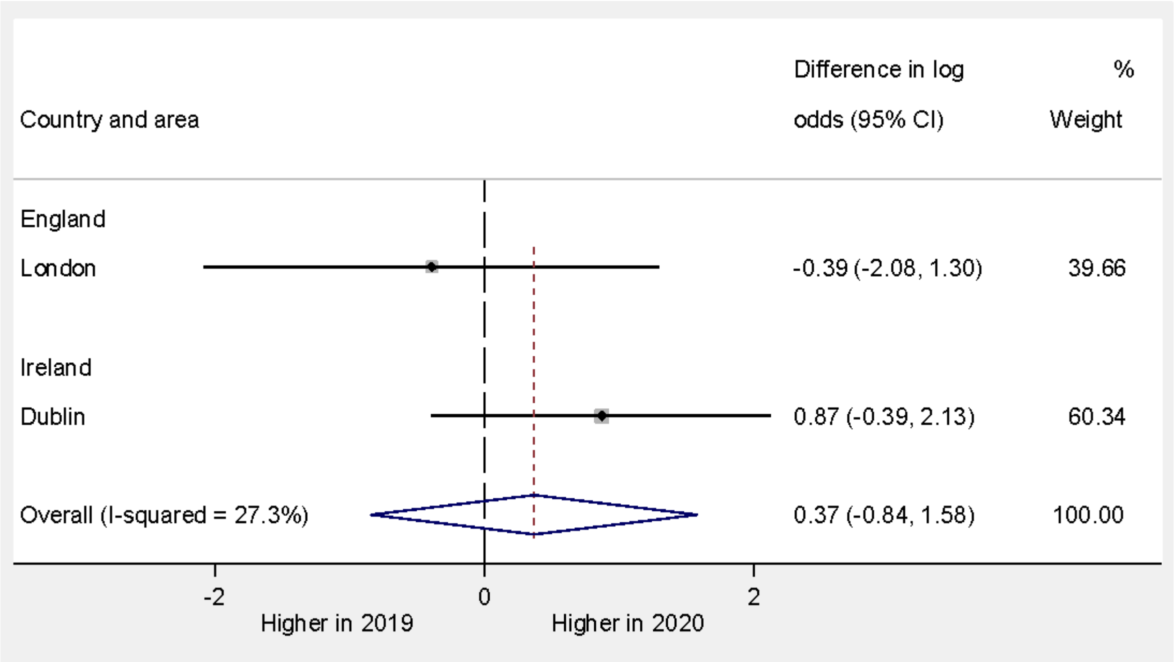

CLINICAL MANAGEMENT OF CHILDREN AND ADOLESCENTS PRESENTING WITH SELF-HARM

Figure 23 Forest plot illustrating year differences in the length of stay in hospital (one day or longer) in children and adolescents presenting with self-harm

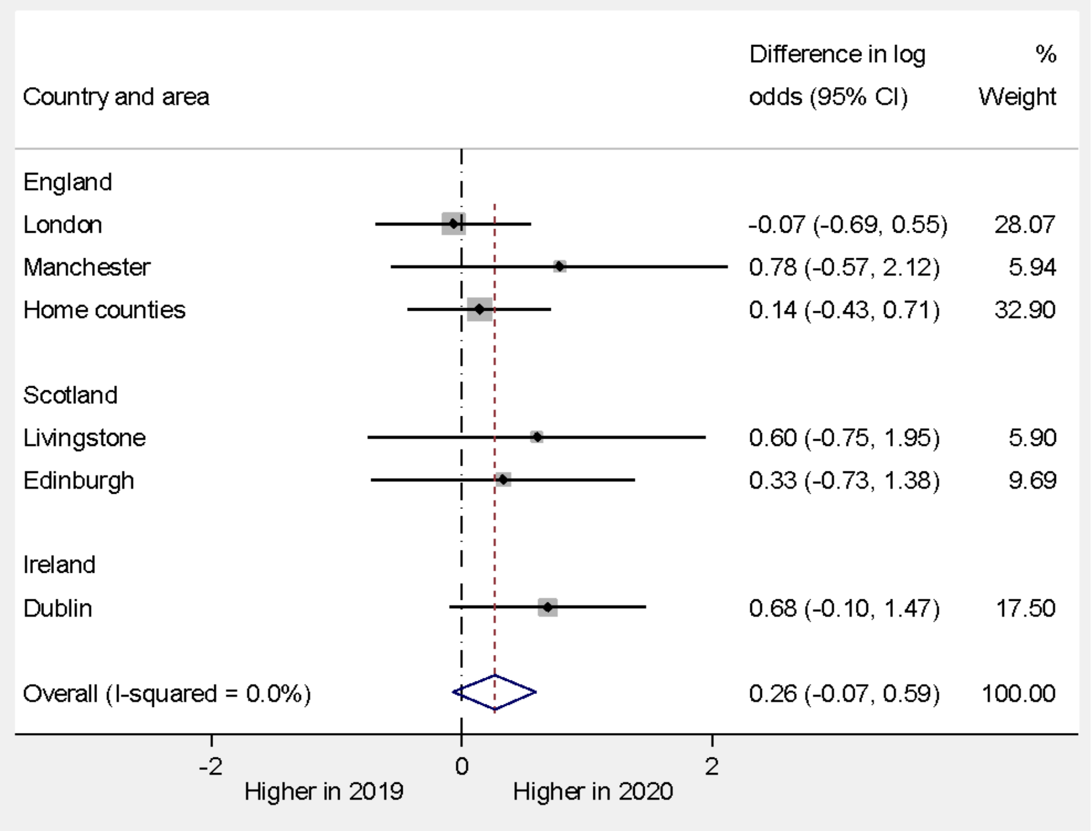

Figure 24 Forest plot illustrating year differences in the proportion of children and adolescents admitted to an acute ward out of all children and adolescents presenting with self-harm

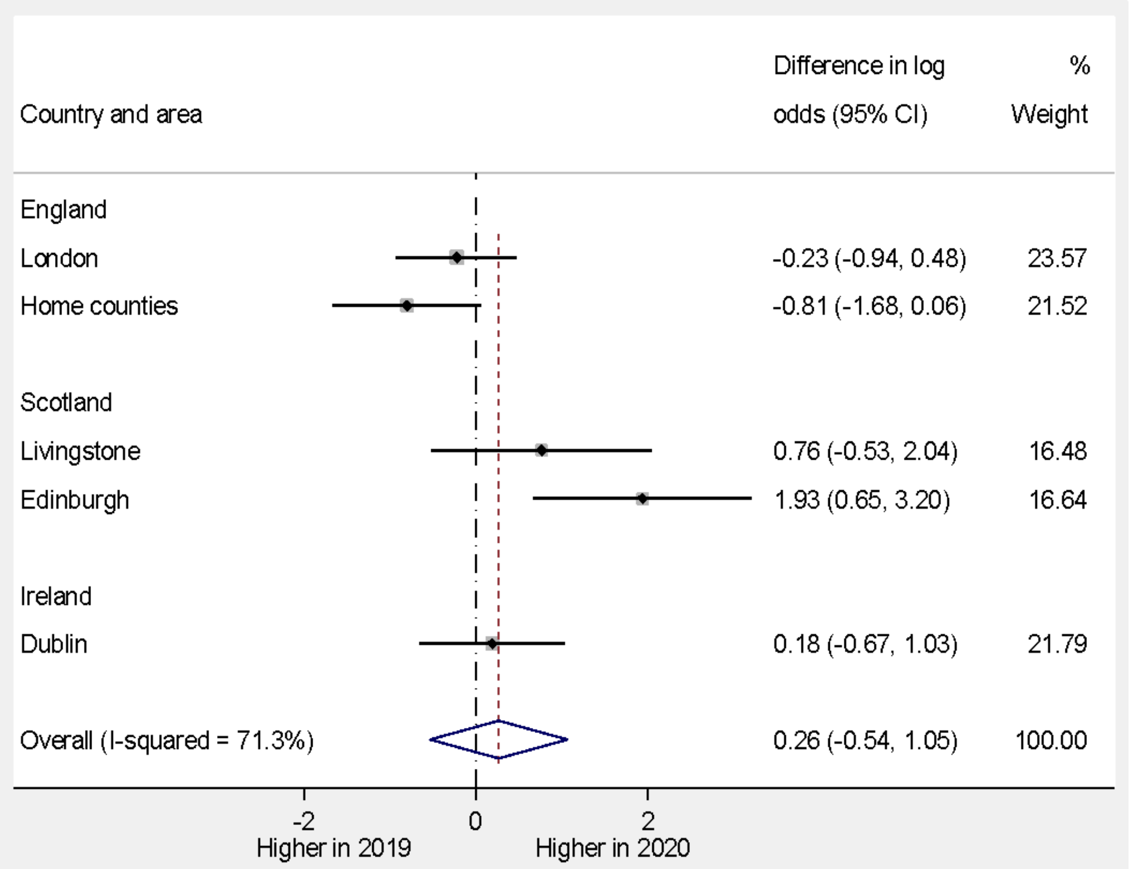

Figure 25 Forest plot illustrating year differences in the proportion of children and adolescents admitted a psychiatric ward out of all children and adolescents presenting with self-harm

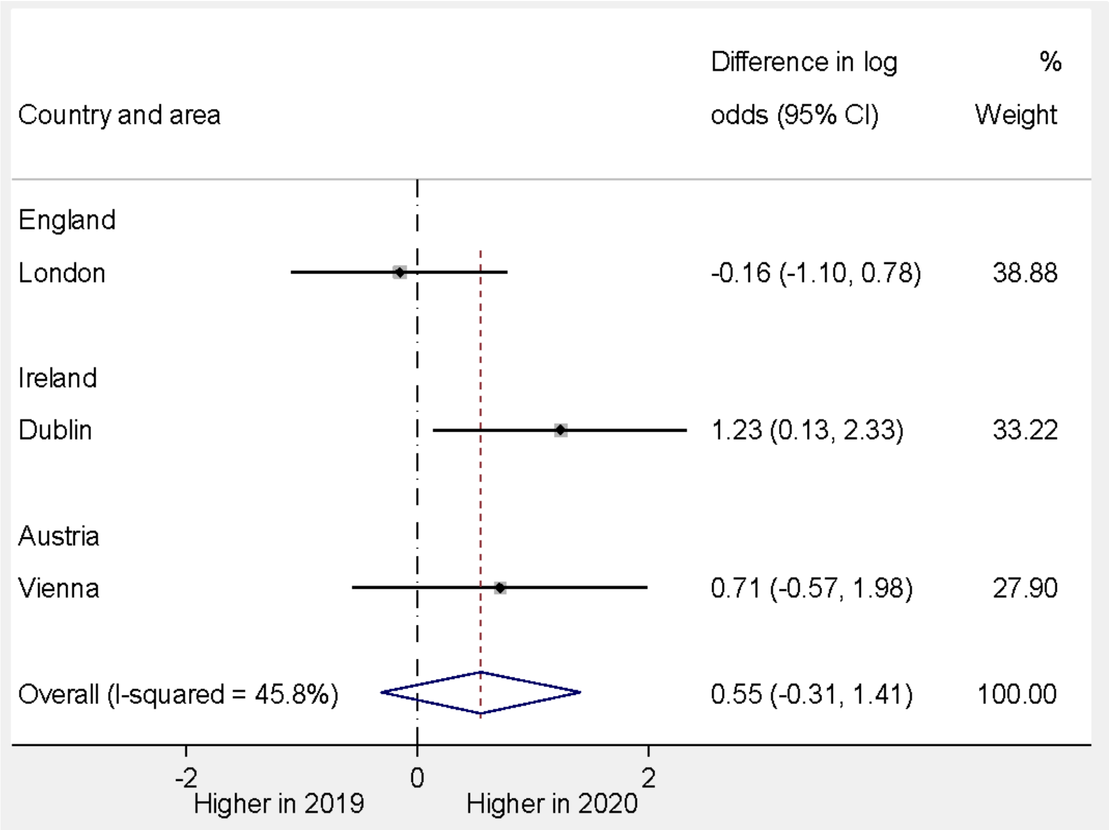

Figure 26 Forest plot illustrating year differences in the proportion of children and adolescents offered community follow-up within seven working days out of all children and adolescents presenting with self-harm

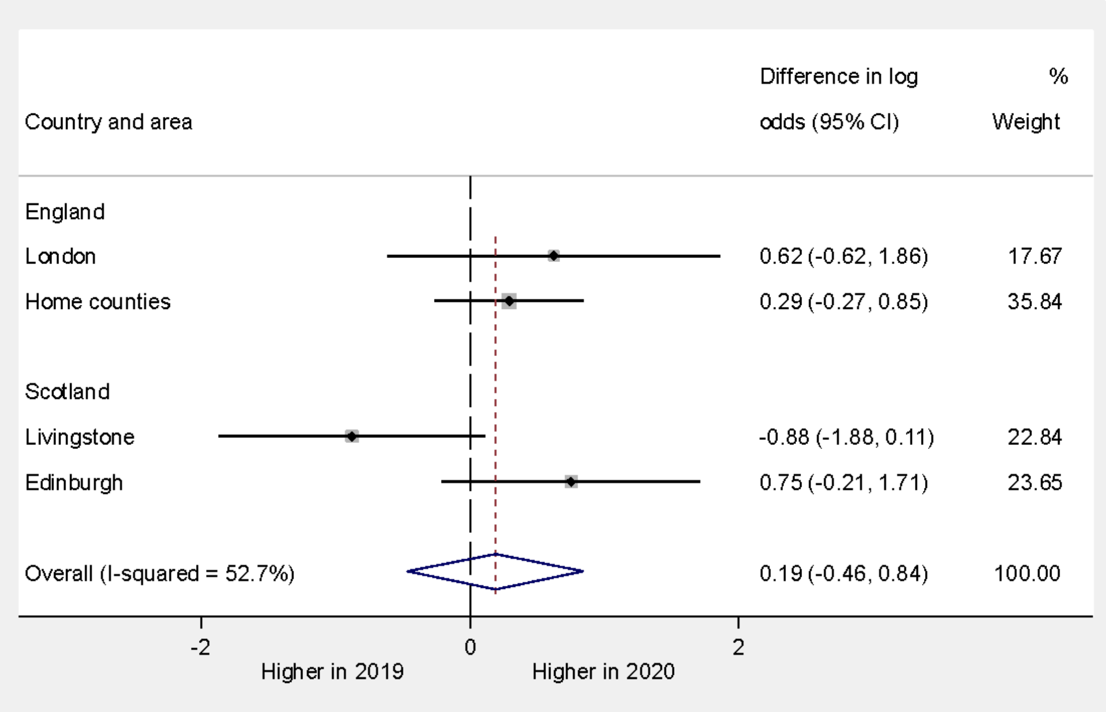

Figure 27 Forest plot illustrating year differences in the proportion of children and adolescents who attended community follow-up within seven working days out of all children and adolescents presenting with self-harm who were offered follow-up

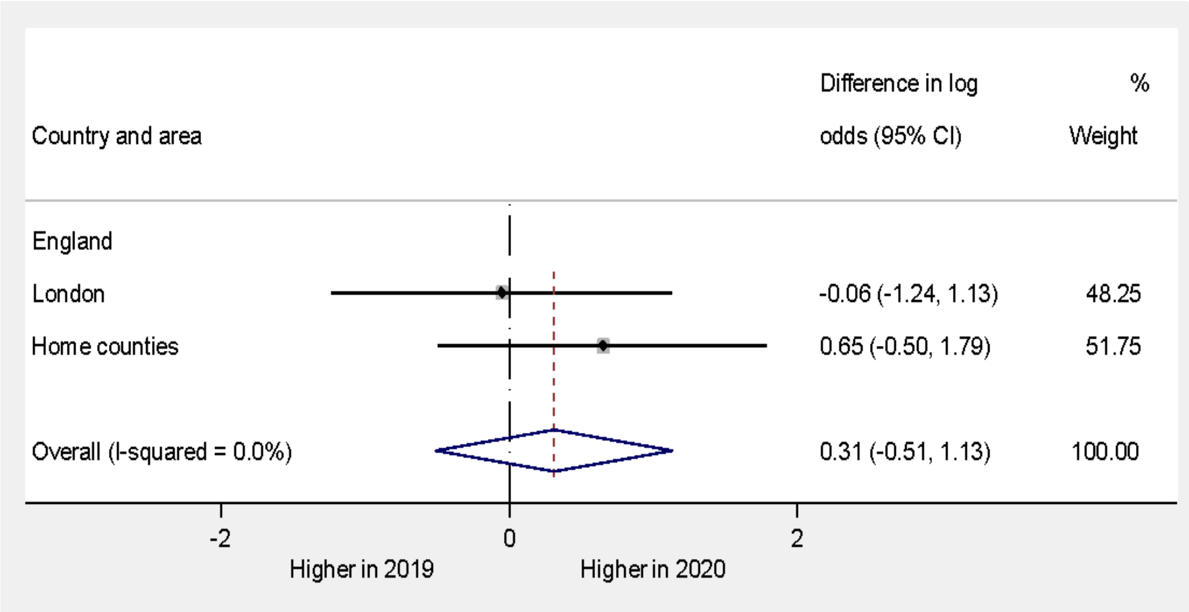

Figure 28 Forest plot illustrating year differences in the proportion of children and adolescents with previous self-harm presentations

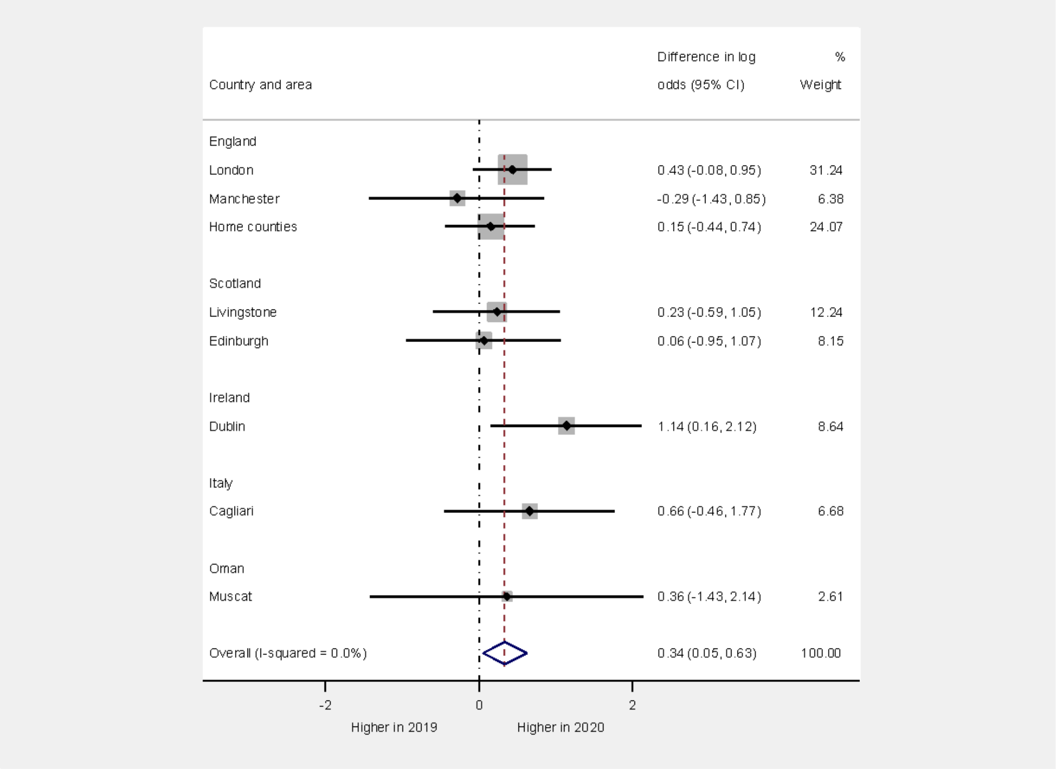

Supplement: Supplementary file 1 — Supplementary file1 (PDF 596 KB) [file 787_2021_1741_MOESM1_ESM.pdf]
